# Supplementary material for: UK medical students’ self-reported knowledge and harm assessment of psychedelics and their application in clinical research: a cross-sectional study
Source: BMJ Open. 2024 Mar 14;14(3):e083595. doi: 10.1136/bmjopen-2023-083595 (PMC10941112; doi:10.1136/bmjopen-2023-083595)
Supplement: Supplementary data [file bmjopen-2023-083595supp001.pdf]

Medical Student Drugs and Mental Health Survey

Start of Block: Participant Information

Q1 **Medical Students' Knowledge and Perceptions of Common "Recreational" Drugs and Their Potential Clinical Uses**

Study Purpose
The study aims to investigate the following:
- Medical students' knowledge and perceptions of commonly used "recreational" drugs.
- Students' perspectives on the current and future clinical uses of these drugs, especially in the treatment of some mental health conditions.
- Factors that influence students' perception of drugs.

The study is led by Marco Schlosser in the UCL Division of Psychiatry and Charlie Song-Smith (BSc), an undergraduate on the MBBS programme at UCL.

Who can participate?
To take part you must be at least 18 years old, have a good understanding of the English language, and be a current student enrolled in a UK medical (MBBS) programme accredited by the General Medical Council (GMC). Students who are currently undertaking an intercalated BSc or other degree as part of their programme (including MBPhD students) are eligible to participate.

What will participation involve?
This study uses an anonymous online survey that approximately 10 minutes to complete. The survey will consist of several brief sections asking about your knowledge, perceptions and some personal factors. After completing the survey, you will have no further involvement and will not be contacted again. To improve the reliability and transparency of scientific research, we will make the fully anonymous data we collect in this study freely available online for other researchers. We greatly appreciate the time you dedicate to this project.

Do I have to participate?
It is up to you to decide whether to take part or not; choosing not to take part will not disadvantage you in any way. If you do decide to take part, you are free to withdraw at any time without giving a reason, by simply closing the browser before the end of the survey. Please note that fully completed surveys cannot be removed as we will be unable to selectively identify and delete your responses from the pool of anonymised data.

What are the possible benefits of taking part?

The data we collect from this study will shed light on how well educated and knowledgeable medical students feel about commonly used drugs, and will therefore help to inform medical education. The study results will also help to gauge students' views on the risks and clinical uses of drugs in relation to mental health, which has potential to influence public policy and future clinical practice. You may personally benefit from an increased knowledge of the various harms of different drugs, as well as increased awareness of the ethical and scientific questions surrounding the clinical uses of some drugs.

Are there any risks of taking part?
The risks to taking part in this study are minimal. Though the survey may touch on sensitive issues concerning illicit drugs, we will not ask you for any information relating to personal use of drugs by yourself or others, nor any other illegal or inappropriate activities. You may feel strongly about your responses, but can rest assured that they may not be linked to you in any way. If you feel distressed or upset by anything in the survey, we encourage you to report this to the researchers via the following:
Mr Marco Schlosser (MSc): marco.schlosser@ucl.ac.uk
Mr Charlie Song-Smith (BSc): charlie.song-smith.19@ucl.ac.uk

Data Protection Privacy Notice
The data you provide is very valuable to us. No personally identifying information will be collected and we will not record your IP address. All obtained data are anonymous. Hence, the publication of study results will not include any data that can directly or indirectly identify you. Your responses will be stored securely by UCL for the duration of the research, putatively for a period of 5 years. University College London (UCL) will act as the data controller for this project. This study constitutes social research which is carried out by the university in the public interest, and your data will be processed in compliance with the UK General Data Protection Regulation (GDPR). This study has received full ethical approval from UCL's Research Ethics Committee (Project ID: 14309/007).

Study results
The results of the study will be analysed, written up and submitted to a peer-reviewed scientific journal. If you would like to know about the results upon completion of the research, irrespective of whether you partake in this study, please follow Marco Schlosser's Open Science Framework profile, which will make all the data and results freely available online: https://osf.io/wgejf/

Thank you for reading this information sheet and for considering whether to take part in this research. Please check the boxes below to confirm that you have read and understood this information sheet and provide your informed consent to participate.

I understand that I am free to withdraw from the study before the completion of the survey. (1)	○ Confirm (1)

I understand that my anonymous responses will be stored securely by UCL for 5 years. (2)	○ Confirm (1)
I am at least 18 years old and have a good understanding of the English language. (3)	○ Confirm (1)
I am currently enrolled as a medical student in a GMC-accredited programme, and I voluntarily consent to participate in this study. (4)	○ Confirm (1)

Skip To: End of Survey If Q1 != I am currently enrolled as a medical student in a GMC-accredited programme, and I voluntarily consent to participate in this study. [Answer 1]

End of Block: Participant Information

Start of Block: Self-reported drug knowledge

Display This Question:

If Q1 = I understand that I am free to withdraw from the study before the completion of the survey. [Answer 1]

And Q1 = I understand that my anonymous responses will be stored securely by UCL for 5 years. [Answer 1]

And Q1 = I am at least 18 years old and have a good understanding of the English language. [Answer 1]

And Q1 = I am currently enrolled as a medical student in a GMC-accredited programme, and I voluntarily consent to participate in this study. [Answer 1]

Q1 Thank you for participating in this survey. Most questions require a single click or tap (if using mobile/tablet) on a sliding visual analogue scale to indicate your response. You are able to move the slider backwards and forwards to fine tune your preferences.

The first section of this questionnaire will focus on your knowledge and perceptions of the following commonly used "recreational" (and illegal) drugs: cannabis, cocaine, heroin, LSD, MDMA (ecstasy) and psilocybin (magic mushrooms).

Firstly, please indicate how well educated/knowledgeable you feel about these drugs.
For example, this would include knowledge of pharmacology, physiology, subjective effects and adverse consequences of the drug.

If you have not heard of the drug in question, please select the checkbox.

	No knowledge	Excellent knowledge	Not heard of it

Cannabis ()	
Cocaine ()	
Heroin ()	
Ketamine ()	
LSD ()	
Magic mushrooms ()	
MDMA (ecstasy) ()	

Q2 How often have you come across the drugs listed above in your teaching, including lectures, small group work and problem-based or self-directed learning?

This excludes any direct observation of patients/members of the public, e.g. on clinical placements.

	Never ... Very often
Cannabis ()	
Cocaine ()	
Heroin ()	
Ketamine ()	
LSD ()	
Magic mushrooms ()	
MDMA (ecstasy) ()	

Song-Smith C, *et al. BMJ Open* 2024; 14:e083595. doi: 10.1136/bmjopen-2023-083595

Q3 How often have you come across these drugs in your own reading/research?

	Never	Very often
Cannabis ()		
Cocaine ()		
Heroin ()		
Ketamine ()		
LSD ()		
Magic mushrooms ()		
MDMA (ecstasy) ()		

End of Block: Self-reported drug knowledge

Start of Block: Perception of drug-related harms

Q4

The following section will ask you to rate a number of different harms associated with the drugs listed, from no harm to severe harm.

When considering these harms, please make your judgments on the assumption that the drugs are pure, i.e. not contaminated with other substances, and taken in isolation. For example, try your best to rate the harms of pure MDMA that is not cut with other drugs, or intentionally mixed with other drugs by the user.

How great is the potential of the drug to cause death by overdose (i.e. the intrinsic lethality)?

No harm Severe harm

0 10 20 30 40 50 60 70 80 90 100

Cannabis ()	
Cocaine ()	
Heroin ()	
Ketamine ()	
LSD ()	
Magic mushrooms ()	
MDMA (ecstasy) ()	

Q5

To what extent is life is shortened by the use of the drug, excluding deaths from overdose? For example, this could include deaths due to road traffic accidents, lung cancers, HIV and suicide.

	No harm										Severe harm
	0	10	20	30	40	50	60	70	80	90	100
Cannabis ()											
Cocaine ()											
Heroin ()											
Ketamine ()											
LSD ()											
Magic mushrooms ()											
MDMA (ecstasy) ()											

Q6
How great is the damage to physical health (short of death) caused by the drug? For example, this could include liver damage, other organ damage, seizures, strokes.

	No harm Severe harm<br>0 10 20 30 40 50 60 70 80 90 100
Cannabis ()	
Cocaine ()	
Heroin ()	
Ketamine ()	
LSD ()	
Magic mushrooms ()	
MDMA (ecstasy) ()	

Q7
To what extent does the drug cause addiction/dependence, i.e. the urge to continue to use despite adverse consequences?

No harm Severe harm

0 10 20 30 40 50 60 70 80 90 100

Song-Smith C, et al. BMJ Open 2024; 14:e083595. doi: 10.1136/bmjopen-2023-083595

Cannabis ()	
Cocaine ()	
Heroin ()	
Ketamine ()	
LSD ()	
Magic mushrooms ()	
MDMA (ecstasy) ()	

Q8
To what extent does the drug cause acute mental health problems when taken?

	No harm	Severe harm
	0 10 20 30 40 50 60 70 80 90 100	
Cannabis ()		
Cocaine ()		
Heroin ()		
Ketamine ()		
LSD ()		
Magic mushrooms ()		
MDMA (ecstasy) ()		

Song-Smith C, *et al. BMJ Open* 2024; 14:e083595. doi: 10.1136/bmjopen-2023-083595

Q9 To what extent does the drug cause problems in the user's work or social life, leading to mental health problems (excluding addiction)?

	No harm	Severe harm
	0 10 20 30 40 50 60 70 80 90 100	
Cannabis ()		
Cocaine ()		
Heroin ()		
Ketamine ()		
LSD ()		
Magic mushrooms ()		
MDMA (ecstasy) ()		

End of Block: Perception of drug-related harms

Start of Block: Drugs in psychiatry

Q10
This short section will ask you for your perceptions of current psychiatric therapies and novel drug treatments.

Thinking about mental health conditions such as anxiety disorders, depression and addiction, how effective would you consider the following general treatment approaches for these conditions?

	Not effective at all	Extremely effective
Pharmaceuticals, e.g. selective-serotonin reuptake inhibitors (SSRIs) ()		
Psychotherapy, e.g. cognitive behavioural therapy (CBT) or counselling ()		

Q11 Consider the following statement: "Drugs such as psilocybin (the active ingredient in magic mushrooms) and MDMA (the active ingredient in "ecstasy") have recently been investigated as potential treatments for mental health conditions such as depression, PTSD and addiction."

Which of the following best describes your own awareness of this research area?

- ◯ I am aware of this, and am well educated on the topic (1)
- ◯ I am aware of this, and curious to know more (2)
- ◯ I am aware of this, but it doesn't interest me (3)
- ◯ I am not aware of this, but am curious to know more (4)
- ◯ I am not aware of this, and it doesn't interest me (5)

Q12 Consider the following statement: "Drugs that are considered to have no medical value and a high risk of misuse or harm are listed in Schedule 1 of the UK Misuse of Drugs Regulations (2001); these currently include MDMA and psilocybin. Research involving schedule 1 drugs is associated with significantly increased costs, duration and difficulty."

Given your current knowledge, to what extent would you support or oppose changing the legal status of these drugs to facilitate further research into their potential medical uses (while keeping restrictions on recreational use as they are)?

	Strongly oppose Strongly support
Psilocybin ()	
MDMA ()	

End of Block: Drugs in psychiatry

Start of Block: Personal factors

Q13
This final section will ask you about some personal factors, including a personality measure and the factors which have influenced your perception of drugs. We appreciate that these questions

may touch on sensitive issues and greatly appreciate your responses.

What year of your course are you in currently?

- 1 (1)
- 2 (2)
- 3 (3)
- 4 (4)
- 5 (5)
- 6 (6)

Q14 Have you previously or are you currently undertaking an intercalated BSc or MBPhD as part of your degree programme?

- Yes (1)
- No (2)

Q15 Have you previously completed a degree (BA/BSc, MSci, PhD, etc) outside of your current programme?

- Yes (1)
- No (2)

Song-Smith C, *et al. BMJ Open* 2024; 14:e083595. doi: 10.1136/bmjopen-2023-083595

Q16 To what extent are you interested in pursuing a career in psychiatry?

- ○ I am confident I will pursue a career in psychiatry (1)
- ○ I consider psychiatry one of my more likely career paths (2)
- ○ I have not ruled out psychiatry, but it is not one of my more likely career paths (3)
- ○ I consider psychiatry one of my less likely career paths (4)
- ○ I am confident I will not pursue a career in psychiatry (5)

Q17 Please rate the importance of the following factors in influencing your perception of drugs (such as those listed in this survey).

	Not important — Very important<br>0 10 20 30 40 50 60 70 80 90 100
My own and my friends' personal experiences ()	
Teaching as part of my course ()	
Media reporting ()	
Reading scientific papers/articles ()	
A drug's legal class (A, B, C) ()	
Advice from parents or other authority figures ()	
General internet searches and online information ()	

Song-Smith C, *et al. BMJ Open* 2024; 14:e083595. doi: 10.1136/bmjopen-2023-083595

Q18 Finally, please select how well you feel the following statements describe your personality.

I see myself as someone who...

	Disagree strongly (1)	Disagree a little (2)	Neither agree nor disagree (3)	Agree a little (4)	Agree strongly (5)
is reserved (1)	○	○	○	○	○
is generally trusting (2)	○	○	○	○	○
tends to be lazy (3)	○	○	○	○	○
is relaxed, handles stress well (4)	○	○	○	○	○
has few artistic interests (5)	○	○	○	○	○
is outgoing, sociable (6)	○	○	○	○	○
tends to find fault with others (7)	○	○	○	○	○
does a thorough job (8)	○	○	○	○	○
gets nervous easily (9)	○	○	○	○	○
has an active imagination (10)	○	○	○	○	○

End of Block: Personal factors

Song-Smith C, *et al. BMJ Open* 2024; 14:e083595. doi: 10.1136/bmjopen-2023-083595
