# Supplementary material for: UK medical students’ self-reported knowledge and harm assessment of psychedelics and their application in clinical research: a cross-sectional study
Source: BMJ Open. 2024 Mar 14;14(3):e083595. doi: 10.1136/bmjopen-2023-083595 (PMC10941112; doi:10.1136/bmjopen-2023-083595)
Supplement: Author's manuscript [file bmjopen-2023-083595.draft_revisions.pdf]

BMJ Open

BMJ Open is committed to open peer review. As part of this commitment we make the peer review history of every article we publish publicly available.

When an article is published we post the peer reviewers' comments and the authors' responses online. We also post the versions of the paper that were used during peer review. These are the versions that the peer review comments apply to.

The versions of the paper that follow are the versions that were submitted during the peer review process. They are not the versions of record or the final published versions. They should not be cited or distributed as the published version of this manuscript.

BMJ Open is an open access journal and the full, final, typeset and author-corrected version of record of the manuscript is available on our site with no access controls, subscription charges or pay-per-view fees (http://bmjopen.bmj.com).

If you have any questions on BMJ Open's open peer review process please email info.bmjopen@bmj.com

BMJ Open

UK medical students' self-reported knowledge and harm assessment of psychedelics and their application in clinical research: a cross-sectional study

Journal:	*BMJ Open*
Manuscript ID	bmjopen-2023-083595
Article Type:	Original research
Date Submitted by the Author:	22-Dec-2023
Complete List of Authors:	Song-Smith, Charlie; University College London, UCL Medical School<br>Jacobs, Edward; University of Oxford, Department of Psychiatry; University of Oxford, Wellcome Centre for Ethics and Humanities<br>Rucker, James; King's College London, The Department of Psychological Medicine; South London and Maudsley NHS Foundation Trust<br>Saint, Matthew; University of Exeter Medical School<br>Cooke, James; Innerspace Institute<br>Schlosser, Marco; UCL, Division of Psychiatry; Institut für Psychotherapie Potsdam
Keywords:	MEDICAL EDUCATION & TRAINING, Cross-Sectional Studies, PSYCHIATRY

SCHOLARONE™
Manuscripts

I, the Submitting Author has the right to grant and does grant on behalf of all authors of the Work (as defined in the below author licence), an exclusive licence and/or a non-exclusive licence for contributions from authors who are: i) UK Crown employees; ii) where BMJ has agreed a CC-BY licence shall apply, and/or iii) in accordance with the terms applicable for US Federal Government officers or employees acting as part of their official duties; on a worldwide, perpetual, irrevocable, royalty-free basis to BMJ Publishing Group Ltd ("BMJ") its licensees and where the relevant Journal is co-owned by BMJ to the co-owners of the Journal, to publish the Work in this journal and any other BMJ products and to exploit all rights, as set out in our licence.

The Submitting Author accepts and understands that any supply made under these terms is made by BMJ to the Submitting Author unless you are acting as an employee on behalf of your employer or a postgraduate student of an affiliated institution which is paying any applicable article publishing charge ("APC") for Open Access articles. Where the Submitting Author wishes to make the Work available on an Open Access basis (and intends to pay the relevant APC), the terms of reuse of such Open Access shall be governed by a Creative Commons licence – details of these licences and which Creative Commons licence will apply to this Work are set out in our licence referred to above.

Other than as permitted in any relevant BMJ Author's Self Archiving Policies, I confirm this Work has not been accepted for publication elsewhere, is not being considered for publication elsewhere and does not duplicate material already published. I confirm all authors consent to publication of this Work and authorise the granting of this licence.

For peer review only

UK medical students' self-reported knowledge and harm assessment of psychedelics and their application in clinical research: a cross-sectional study

Charlie Song-Smith[a, 1], Edward Jacobs[b, c], James Rucker[d, e], Matthew Saint[f], James Cooke[g], & Marco Schlosser[h, i, 1]

[a] UCL Medical School, University College London, London, United Kingdom
[b] Department of Psychiatry, University of Oxford, Oxford, United Kingdom
[c] Wellcome Centre for Ethics and Humanities, University of Oxford, Oxford, United Kingdom
[d] The Department of Psychological Medicine, Institute of Psychiatry, Psychology & Neuroscience, King's College London, London, United Kingdom
[e] South London & Maudsley NHS Foundation Trust, Monks Orchard Road, Beckenham, Kent, United Kingdom
[f] University of Exeter Medical School, Exeter, UK
[g] Innerspace Institute, Portugal
[h] Division of Psychiatry, Faculty of Brain Sciences, University College London, London, United Kingdom
[i] Institut für Psychotherapie Potsdam, Potsdam, Germany

Marco Schlosser and James Cooke share last authorship.

[1] Corresponding authors: Charlie Song-Smith & Marco Schlosser

Charlie Song-Smith,
Email: charlie.song-smith.19@ucl.ac.uk
UCL Medical School
University College London
London, United Kingdom

Marco Schlosser
Email: marco.schlosser@ucl.ac.uk
Division of Psychiatry
University College London
London, United Kingdom

Funding statement: This research received no specific grant from any funding agency in the public, commercial or not-for-profit sectors. James Rucker received salary support via the NIHR Clinician Scientist Fellowship Programme during the period of this research (CS-2017-17-007).

Competing interests statement: James Rucker has received payments for advisory work from Clerkenwell Health (Past), Beckley PsyTech (Past), Delica Therapeutics (Past), for articles written for Janssen, and financial assistance for attendance at conferences from Compass Pathways (past) and Janssen. James Rucker has received grant funding (received and managed by King's College London) from Compass Pathfinder, Beckley PsyTech, Multidisciplinary Association for Psychedelic Studies, National Institute for Health Research, Wellcome Trust, Biomedical Research Centre at the South London and Maudsley NHS Foundation Trust. All other authors declare that they have no competing interests.

For peer review only - http://bmjopen.bmj.com/site/about/guidelines.xhtml

Abstract

Objective: To capture UK medical students' self-reported knowledge and harm assessment of psychedelics and to explore the factors associated with support for changing the legal status of psychedelics to facilitate further research.

Design: Cross-sectional, anonymous online survey of UK medical students using a non-random sampling method.

Setting: UK medical schools recognised by the General Medical Council.

Participants: The sample consisted of 132 medical students who had spent an average of 3.8 years (SD=1.4; range: 1 to 6) in medical school.

Results: Most students (83%) reported that they were aware of psychedelic research and only four participants (3%) said that they were not interested in learning more about this type of research. Although medical students' harm assessment of psychedelics closely aligned with that of experts, only 17% of students felt well-educated on psychedelic research. Teachings on psychedelics were only rarely encountered in their curriculum. Time spent at medical schools was not associated with more knowledge about psychedelics. On average, this sample of medical students showed strong support for changing the legal status of psychedelics to facilitate further research into their potential clinical applications. Regression modelling indicated that greater knowledge of psychedelics, lower estimated harm scores, more time spent in medical school, and lower perceived effectiveness of nonpharmacological mental health treatments were associated with a support for legal status change.

Conclusions: This is the first study to explore UK medical students' self-reported knowledge and attitudes towards psychedelics and their application in clinical research. Our findings reveal a significant interest among medical students to learn more about psychedelic research and a strong support for further psychedelic research. Future studies are needed to examine how medical education could be refined to adequately prepare medical students for a changing healthcare landscape in which psychedelic-assisted therapy could soon be implemented in clinical practice.

Keywords: Medical students, Psychedelics, Knowledge, Harm assessment, Medical education

Word count: 4618

For peer review only

Strengths and limitations of this study

- This study presents the first survey of UK medical students' attitudes towards psychedelics and their application in clinical research.
- The wide-ranging assessment included medical students' self-reported knowledge of psychedelics, a multidimensional harm assessment of psychedelics, level of support for changing the legal status of psychedelics to facilitate further research into their potential clinical applications, and students' perceptions of medical education on psychedelics and psychedelic research.
- The cross-sectional nature of our data prevented us from drawing causal conclusions about potential changes in students' attitudes and knowledge over time.
- The limited range of demographic variables did not allow for an in-depth characterisation of this sample and important predictors of attitudes might have been missed. Non-random sampling and selection bias could have impacted the generalisability of our findings as students already interested in drugs might have been more likely to participate.

For peer review only

Introduction

Since the early 1990s, there has been a steady resurgence of research involving psychedelics such as lysergic acid diethylamide (LSD), psilocybin, and N, N-Dimethyltryptamine (DMT) (1–3). After a short-lived period of medical use of psychedelics by clinicians and researchers in the middle of the 20th century, psychedelic research was effectively ended by prohibitive legislature in various countries (4). The clinical trials conducted prior to these legal sanctions suggested that psychedelics – which were frequently used in combination with or as an aid to psychotherapy – could positively impact a range of disorders, particularly mood disorders and alcohol dependence (5,6). These early findings have been corroborated by modern-era clinical trials, which support the therapeutic effects of psychedelics and psychedelic-assisted psychotherapy in some patient groups (for reviews see e.g, (7–9)). The largest published and ongoing psychedelic trials investigate the effects of psilocybin in combination with psychological support for the treatment of major depressive disorder (e.g, (10–14)). Clinical investigations into the therapeutic efficacy of related drugs, such as 3,4-Methylenedioxymethamphetamine (MDMA) for post-traumatic stress disorder (PTSD) (15,16) and ketamine for depression (17–19), have also advanced substantially. In the context of the present study, the term "psychedelic" will encompass the classic psychedelics (e.g., psilocybin, LSD) as well as MDMA and ketamine.

If current developments continue unhindered, some psychedelics and forms of psychedelic-assisted therapy will likely receive regulatory approval and thus transition from legally restricted use in research studies to integration in clinical practice (but see (20)). The extent to which psychedelics will be clinically implemented and disseminated will also depend on medical professionals' knowledge and attitudes towards these drugs. Despite the looming possibility of regulatory approval, research on medical professionals' opinions surrounding psychedelic and their use in clinical practice is in its infancy. A recent survey

For peer review only

indicated that US psychiatrists (n=106) largely believed in the therapeutic potential of psychedelics and were strongly in favour of federal funding to facilitate further research with these substances (21). Variables associated with a favourable assessment of psychedelics included greater psychedelic knowledge, fewer concerns about the drugs' addictive potential, and working in research. A prior survey study led by the same author had also suggested that psychiatrists (n=324) were supportive of further psychedelic research on mental health disorders, but the sample also tended to view these drugs as potentially dangerous and agreed with their restrictive scheduling for recreational use (22). A pilot study (n=83) of psychiatrists working in the UK's National Health Service (NHS) showed that most of them (77%) believed that controlled or therapeutic use of psychedelics should play a role in society (23). Notably, psychiatrists of all grades reported significant training needs and lacking preparedness or confidence to deliver psychedelic-assisted psychotherapy should it become available. Overall, most research conducted in this nascent field converges on the conclusion that a majority of psychiatrists and other mental health professionals – including psychologists and counsellors (24,25) – are supportive of further clinical research with psychedelics.

Given that tomorrow's psychiatrists are today's medical students, it is pertinent to consider medical students' knowledge and attitudes towards psychedelics. To date, only three studies on this topic have been published (26–28). In a sample of 213 US medical students, 83% and 52% of respondents believed that psilocybin should be legalised for recreational use and medical use, respectively (26). Students with greater self-reported knowledge of psilocybin and fewer concerns about potential negative effects reported more positive overall perceptions regarding the medical use of psilocybin. Another survey study of US medical students (n=132) suggested that almost all respondents (95%) believed that further research examining psychedelics' therapeutic potential is warranted (27). Overall, medical students

For peer review only - http://bmjopen.bmj.com/site/about/guidelines.xhtml

held positive attitudes toward psychedelics, but only 41% reported that they felt knowledgeable about psychedelics. None of the demographic variables, including age, sex, planned specialty (i.e., psychiatry, internal medicine etc.), and level of training, were associated with greater positive attitudes towards psychedelics. Lastly, another survey of US medical students (n=295) indicated that more favourable attitudes towards psychedelics were associated with a greater willingness to recommend psychedelic-assisted therapy to patients should it receive FDA approval (28). The present study aimed to further expand knowledge in this area by sampling the views and attitudes of medical students in the United Kingdom.

Using an anonymous online survey, we aimed to capture UK medical students' self-reported knowledge of psychedelics and their application in clinical research. We also aimed to compare medical students' harm assessment of psychoactive drugs with that of drug experts. We also aimed to examine to which extent participant characteristics were associated with support for changing the legal status of psychedelics to facilitate further research. Given the lack of empirical and theoretical work in this area, our hypotheses and statistical approaches should be viewed as primarily exploratory and our findings as preliminary. We expected more time spent in medical school to be associated with higher psychedelic knowledge and lower psychedelic harm composite scores. Regarding support for changing the legal status of psychedelics to facilitate further research, we expected students more knowledgeable of psychedelics to be more supportive of their legal status change; students assigning lower harm to psychedelics to be more supportive; more seasoned students to be more supportive; more psychiatrically interested students to be more supportive; students favourably assessing pharmacological and nonpharmacological to be more and less supportive, respectively; students more open to new experiences to be more supportive; and students with higher levels of neuroticism to be less supportive.

For peer review only

For peer review only - http://bmjopen.bmj.com/site/about/guidelines.xhtml

Methods

Procedures and survey design

This study used an anonymous online survey delivered via the Qualtrics web platform to collect cross-sectional data without recording participants' IP addresses. A short text asking individuals whether they would like to support research on medical students' knowledge and perceptions of common "recreational" drugs and their clinical uses was shared on UK university medical society mailing lists and bulletins, and social media platforms (LinkedIn, Twitter). To reduce self-selection bias, the study was advertised as a "Drugs and Mental Health" survey, rather than a survey about psychedelics. Interested individuals could follow a link to the study information sheet, which described the aims of the survey, the anonymous data collection, and the eligibility criteria (i.e. at least 18 years old, good understanding of the English language, currently enrolled in a UK medical programme accredited by the General Medical Council). Individuals were required to indicate their informed consent before starting the survey. The survey took approximately 10 minutes to complete and was accessible between October 2021 and March 2022. No financial or other incentives were offered for participation. This study received ethical approval from University College London's Research Ethics Committee (Project ID: 14309/007) and was performed in accordance with the 1964 Declaration of Helsinki and its later amendments. All data was collected and processed in accordance with the 2018 UK General Data Protection Regulation. Patients or the public were not involved in the design, conduct, reporting, or dissemination plans of our research.

Participants

A total of 136 individuals consented to participate and completed the survey. Incomplete responses were automatically deleted by Qualtrics. In the service of survey

For peer review only

quality control, we excluded four participants who took less than 5 minutes to complete the survey (i.e., the time it took to read through the survey at a fast pace without responding to any questions). The total sample thus consisted of medical 132 students without missing data.

Measures

To ensure anonymity, we collected only minimal demographic information including course year (i.e., years since starting training), participation in intercalated BSc or MBPhD programme, previous completion of university degree, and interest in pursuing a career in psychiatry.

To capture medical students' self-reported knowledge and the amount of education they had received on seven drugs (i.e, cannabis, cocaine, heroin, ketamine, LSD, magic mushrooms/psilocybin, ecstasy/MDMA), we used the following three statements:

1. Self-reported knowledge: "Firstly, please indicate how well educated/knowledgeable you feel about these drugs. For example, this would include knowledge of pharmacology, physiology, subjective effects and adverse consequences of the drug."
2. Encountered in curriculum: "How often have you come across the drugs listed above in your teaching, including lectures, small group work and problem-based or self-directed learning?"
3. Encountered in extracurricular reading: "How often have you come across these drugs in your own reading/research?"

The first statement used a sliding visual analogue scale (VAS) ranging from 0 ('no knowledge') to 100 ('excellent knowledge'), whilst the latter two questions used a VAS from 0 ('never') to 100 ('very often'). Commonly used non-psychedelic drugs were included to allow comparison of relative perceived harms, as well as to cover the fact that this was primarily a psychedelic survey. A psychedelic knowledge composite was computed by averaging the knowledge scores of LSD, psilocybin, MDMA, and ketamine (i.e., four scale

For peer review only - http://bmjopen.bmj.com/site/about/guidelines.xhtml

scores were combined), with higher composite scores indicating greater self-reported knowledge.

To measure medical students' perceptions of harms of the same seven drugs, the following six questions with a VAS from 0 (no harm) to 100 (severe harm) were used:

4. Drug-specific mortality: "How great is the potential of the drug to cause death by overdose (i.e., the intrinsic lethality)?"
5. Drug-related mortality: "To what extent is life shortened by the use of the drug, excluding deaths from overdose? For example, this could include deaths due to road traffic accidents, lung cancers, HIV and suicide."
6. Drug-specific damage: "How great is the damage to physical health (short of death) caused by the drug? For example, this could include liver damage, other organ damage, seizures, strokes."
7. Dependence: "To what extent does the drug cause addiction/dependence, i.e., the urge to continue to use despite adverse consequences?"
8. Drug-specific impairment of mental functioning: "To what extent does the drug cause acute mental health problems when taken?"
9. Drug-related impairment of mental functioning: "To what extent does the drug cause problems in the user's work or social life, leading to mental health problems (excluding addiction)?"

A psychedelic harm composite score was computed by averaging the harm scores of LSD, psilocybin, MDMA, and ketamine across the six harm assessment categories (i.e., 24 scale scores were combined), with higher composite scores indicating higher levels of harm. The harm-related questions were adopted from a cross-sectional survey (30) that utilised the 16 harm criteria introduced by a multidisciplinary panel of drug experts in a seminal paper on relative drug harms on behalf of the in the UK's Independent Scientific Committee on Drugs

For peer review only

(29). Using a brief survey to minimise attrition rate, we adopted only six of the 16 criteria covering physical harm (drug-specific mortality, drug-related mortality, drug-specific damage) and psychological harm (dependence, drug-specific impairment of mental functioning, drug-related impairment of mental functioning). Detailed information about these harm criteria can be found Nutt et al. (29).

To capture medical students' attitudes towards mental health treatments and research on psychedelics, we used the following questions and scales:

10. Perception of effectiveness of pharmacological (e.g., selective-serotonin reuptake inhibitors [SSRIs] and nonpharmacological treatments (e.g., psychotherapy): "Thinking about mental health conditions such as anxiety disorders, depression and addiction, how effective would you consider the following general treatment approaches for these conditions?" (VAS from 0 [not effective at all] to 100 [extremely effective])
11. Awareness of and interest in psychedelic research (adapted from the Psychedelic as Medicine Report, (31)): "Consider the following statement: 'Drugs such as psilocybin (the active ingredient in magic mushrooms) and MDMA (the active ingredient in ecstasy) have recently been investigated as potential treatments for mental health conditions such as depression, PTSD, and addiction.' Which of the following best describes your own awareness of this research area?" (5-point nominal scale: I am aware of this, and am well educated on the topic, I am aware of this, and curious to know more; I am aware of this, but it doesn't interest me; I am not aware of this, but am curious to know more; I am not aware of this, and it doesn't interest me).
12. Attitude towards changing legal status of psilocybin and MDMA: "Consider the following statement: 'Drugs that are considered to have no medical value and a high

For peer review only - http://bmjopen.bmj.com/site/about/guidelines.xhtml

risk of misuse or harm are listed in Schedule 1 of the UK Misuse of Drugs Regulations (2001); these currently include MDMA and psilocybin. Research involving schedule 1 drugs is associated with significantly increased costs, duration, and difficulty.' Given your current knowledge, to what extent would you support or oppose changing the legal status of these drugs to facilitate further research into their potential medical uses (while keeping restrictions on recreational use as they are)?" (VAS from 0 [strongly oppose] to 100 [strongly support])

A 'legal change' composite variable was created by averaging the psilocybin and MDMA legal change scores, with higher scores indicating greater support for changing the legal status to facilitate further research.

Another drug-related question asked medical students to rate, using a VAS from 0 (not important) to 100 (very important), the importance of the following factors in influencing their perception of the drugs included in this survey: my own and my friends' personal experiences; teaching as part of my course; media reporting; reading scientific papers/articles; a drug's legal class; advice from parents or other authority figures; general internet searches and online information.

To measure personality traits, the short version of the Big Five Inventory was used (32). This 10-item scale uses a 5-point Likert scale ranging from 1 (disagree strongly) to 5 (agree strongly) to capture openness to experience, conscientiousness, extraversion, agreeableness, and neuroticism (33). Each personality trait is captured by two items and total scores for each trait are computed by averaging the respective items scores. Higher subscale scores relate to higher trait levels. The 10-item version, despite being significantly shorter than the standard 44-item Big Five Inventory on which it is based, has retained good levels of reliability and validity (32).

Data analysis

For peer review only - http://bmjopen.bmj.com/site/about/guidelines.xhtml

Associations of drug-related measures with course year

The relationship between course year and drug-related measures was assessed using Pearson's correlation coefficients.

Regression analysis

We built the statistical models within a risk prediction framework using a stepwise backward elimination approach. The full mode considered the legal change composite score as the continuous outcome. Given the lack of research on medical students' attitudes towards psychedelic research that could guide the selection of potential predictors, we decided to include the following variables as continuous explanatory variables: psychedelic knowledge composite scores, psychedelic harm composite scores, course year, interest in psychiatry, perception of effectiveness of mental health treatments, openness to experience, and neuroticism. The explanatory variable associated with the highest p-value above 0.05 was then deleted. The model was then refitted without the deleted explanatory variable and the estimates and their associated p-values recomputed. The final prediction model thus retained explanatory variables if they were associated with $p < 0.05$. This threshold for inclusion was chosen to ensure model stability and reduce overfitting. Stata/MP version 16.0 and R version 4.0.2 were used for all analyses.

Data availability statement

All data and analysis scripts can be found on the senior author's Open Science Framework profile: https://osf.io/tmp3u/.

Results

Descriptive statistics

Participant characteristics for the total sample of 132 medical students are displayed in Table 1. Descriptive statistics of self-reported knowledge and the extent to which medical students had encountered a discussion of the listed drugs (i.e., cannabis, cocaine, heroin,

For peer review only - http://bmjopen.bmj.com/site/about/guidelines.xhtml

ketamine, LSD, psilocybin, MDMA) in their curriculum or during extracurricular reading and research are displayed in Table 2. Descriptive statistics on medical students' assessment of the harm caused by the listed drugs are reported in Table 3 and visually depicted in Figure 1.

The mean rating (on a scale from 0 [strongly oppose] to 100 [strongly support]) for the change in legal status for psilocybin was 80.2 (SD = 24.8) and for MDMA was 74.3 (SD = 27.7). Both distributions were left-skewed (i.e., more values are on the right side of the mean) with 59 (44.7%) and 50 (37.9%) medical students having a score of 100 for psilocybin and MDMA, respectively. The legal change composite score showed a similar divergence from normality (skewness: -0.98, kurtosis: 3.02).

The other two composite scores did not markedly diverge from normality as indicated by estimates of skewness (psychedelic knowledge: -0.17; psychedelic harm: -0.13) and kurtosis (psychedelic knowledge: 2.30; psychedelic harm: 2.34) and visual inspection of the histograms.

Associations of drug-related measures with course year

More course years were associated with lower psychedelic harm composite scores (Pearson's correlation coefficient r = -0.22, 95% CI: -0.05 to -0.38, p = 0.012). In contrast to our expectations, number of course years were not correlated with higher psychedelic knowledge composite scores (r = 0.12, 95% CI: -0.04 to 0.30, p = 0.129).

Regression analysis

Table 4 reports the results from the full model and the final prediction model. Using a stepwise backward elimination approach with significance threshold of $p = 0.05$, explanatory variables were removed in the following order: neuroticism, perception of pharmacological treatments, interest in psychiatry, and, in the last step, openness to experience. The final prediction model for legal change composite scores thus retained psychedelic knowledge composite scores, psychedelic harm composite scores, course year, and perception of the

For peer review only - http://bmjopen.bmj.com/site/about/guidelines.xhtml

effectiveness of nonpharmacological treatments (all unstandardised estimates are displayed in Table 4). In other words, in the final regression model, greater psychedelic knowledge and more course years were associated with greater support for legal change, whereas greater psychedelic harm scores and a more favourable perception of the effectiveness of nonpharmacological treatments were associated with weaker support for legal change of psychedelics. Assumptions for all regression models were met.

Discussion

The primary aim of this cross-sectional study was to capture UK medical students' self-reported knowledge and harm assessment of psychedelics and the degree to which they would support changing the legal status of psychedelic substances, particularly psilocybin and MDMA, to facilitate further research into their potential medical uses without changing restrictions on recreational use. Results indicated that 83% of medical students were aware of psychedelic research and only four participants said that they were not interested in learning more about this type of research. Medical students evaluated their own experience and that of their friends as the most important influence on their perception of drugs in general. Another important influence was their study of scientific articles, whereas media reporting and a drug's legal class had the least influence on their perception of drugs.

UK medical students were highly supportive of changing the legal status of psilocybin and MDMA, with 45% and 38% expressing the strongest possible support (i.e., a score of 100 on a scale from 0 [strongly oppose] to 100 [strongly support]) for psilocybin and MDMA, respectively. Relatedly, we aimed to explore the variables that would predict the extent to which medical students were in support of changing the legal status. Regression modelling suggested that students who reported greater knowledge of psychedelics, assigned lower harm scores to psychedelics, had spent more time spent in medical education, and assigned lower effectiveness scores to nonpharmacological mental health treatments showed

For peer review only

a greater support for legal status change. The contribution of these variables was in line with our prediction. However, diverging from our expectation was the absence of a relationship between support for legal change and an interest in psychiatry, the perceived effectiveness of pharmacological treatments, and the personality traits of openness to experience and neuroticism.

Although time spent in medical school itself was associated with lower harm ratings of psychedelics, it was not associated with greater self-reported knowledge of psychedelics, suggesting that medical degree curricula do not contain much teaching about psychedelics. This explanation also aligns with the low reported frequency with which medical students encountered the classic psychedelics, psilocybin and LSD, in their curriculum. An alternative explanation, for instance, that medical students attracted to a survey on drugs and mental health tend to have greater knowledge of psychedelics (resulting in ceiling effects), was not reflected in the data, which showed self-reported psychedelic knowledge to be approximately normally distributed.

Medical students' multidimensional harm assessment of seven drugs resulted in an order of assigned overall harm scores that closely matched the order that was assigned by drug expert in a widely-cited harm assessment report conducted on behalf of the UK's Independent Scientific Committee on Drugs (29). In alignment with the expert rating, medical students assigned the highest overall harm score to heroin and cocaine and the lowest to psilocybin. Differences emerged in relation to the rank order of cannabis, which experts rated as substantially more harmful than medical students. In contrast, medical students perceived MDMA to be the third most harmful substance amongst the list of seven drugs assessed in the present study, whereas experts judged the harm of MDMA to be similar to that of LSD and psilocybin.

Limitations

For peer review only - http://bmjopen.bmj.com/site/about/guidelines.xhtml

This study has important limitations that need to be considered when interpreting our results. First, the cross-sectional nature of our data prevents us from drawing causal conclusions about the association between support for changing the legal status of psychedelics and the potential predictors we have considered. Future research with larger sample sizes, more detailed demographic information, objective assessments of students' knowledge, and longitudinal follow-ups are needed to further understand how medical education shapes students' perception of psychedelics and the factors that influence the degree to which students' views align with empirical evidence and expert opinion. Second, some of the measures we applied were tailor-made for this survey and not previously validated. Our harm assessment was adapted from Nutt et al. (29), but we used only six of the 16 harm criteria these researchers proposed; and none of our harm criteria captured harm to others. We also did not include an assessment of other commonly used drugs, such as alcohol and tobacco, preventing us from drawing potentially insightful comparisons. The brevity of our assessment was to keep attrition rates as low as possible. Future research would benefit from a more in-depth and comprehensive assessment of UK medical students' views and attitudes surrounding psychedelics and their application in clinical research. Third, we relied on self-report data gathered via an anonymous online survey; we did not conduct an objective assessment of knowledge about psychedelics (e.g., a multiple choice test) and, therefore, cannot assess the validity and reliability of students' self-report. Lastly, generalisability of our study may be limited due to the present sample's high awareness of and interest in psychedelic research. Despite efforts to minimise selection bias by avoiding the term "psychedelic" during recruitment, it is likely that the students who completed the survey were not representative of the general UK medical student population.

Conclusion

For peer review only

This is the first study to explore UK medical students' self-reported knowledge and perceptions of psychedelics. Medical students showed strong support for changing the legal status of psychedelics to facilitate further research into their potential clinical applications. In general, medical students only rarely encountered teachings on psychedelics in their medical education. Whilst only a small percentage of medical students felt well-educated on psychedelic research, a large majority were interested to learn more, likely reflecting the re-emergence of clinical interest in this area.

Author contributions

Charlie Song-Smith: Conception of the work, Methodology, Data acquisition, Data analysis, Reviewing the work critically for important intellectual content.

Edward Jacobs: Conception of the work, Methodology, Reviewing the work critically for important intellectual content, Supervision.

James Rucker: Conception of the work, Reviewing the work critically for important intellectual content.

Matthew Saint: Conception of the work, Reviewing the work critically for important intellectual content.

James Cooke: Conception of the work, Methodology, Reviewing the work critically for important intellectual content, Supervision.

Marco Schlosser: Writing of the original draft, Conception of the work, Methodology, Data analysis, Data interpretation, Visualisation, Supervision.

For peer review only - http://bmjopen.bmj.com/site/about/guidelines.xhtml

Table 1. Demographics and participant characteristics (*N* = 132).

Variable	Mean (SD) or *n* (%)
Course years (i.e., time spent in medical education)	3.8 (1.4), range: 1 to 6
First year	6 (4.6%)
Second year	14 (10.6%)
Third year	40 (30.3%)
Fourth year	32 (24.2%)
Fifth year	17 (12.9%)
Sixth year	23 (17.4%)
Enrolled in or completed an intercalated degree	62 (47.0%)
Attained a previous university degree	29 (22.0%)
Interest in psychiatry	
I am confident I will pursue a career in psychiatry.	8 (6.1%)
I consider psychiatry one of my more likely career paths.	35 (26.5%)
I have not ruled out psychiatry, but it is not one of my more likely career paths.	39 (29.6%)
I consider psychiatry one of my less likely career paths.	35 (26.5%)
I am confident I will not pursue a career in psychiatry.	15 (11.4%)
Influence on perceptions of drugs (range: 0 [not important] to 100 [very important])	
My own and my friends' personal experiences	77 (20)
Teaching as part of my course	44 (29)
Media reporting	39 (26)
Reading scientific papers/articles	65 (28)
A drug's legal class	28 (29)
Advice from parents or other authority figures	34 (28)
General internet searches and online information	61 (24)
Awareness of and interest in psychedelic research	
I am aware of this, and am well educated on the topic.	22 (16.7%)
I am aware of this, and curious to know more.	84 (63.6%)
I am aware of this, but it doesn't interest me.	3 (2.3%)
I am not aware of this, but am curious to know more.	22 (16.7%)
I am not aware of this, and it doesn't interest me.	1 (0.8%)
Perception of mental health treatments (0 [not effective] to 100 [extremely effective])	
Pharmacological (e.g., selective-serotonin reuptake inhibitors [SSRIs])	58.7 (19.7)
Psychotherapy	66.9 (20.9)
Openness to experience (BFI-10)	3.72 (0.92)
Conscientiousness (BFI-10)	3.76 (0.88)
Extraversion (BFI-10)	3.61 (0.98)
Agreeableness (BFI-10)	3.63 (0.86)
Neuroticism (BFI-10)	3.01 (1.14)

Abbreviations: SD, standard deviation; BFI-10, 10-item Big Five Inventory.

For peer review only

Table 2. Descriptive statistics of medical students' self-reported knowledge and frequency of encountering psychedelics in teaching and in extracurricular reading ($N = 132$).

	Self-reported knowledge [1]	Encountered in curriculum [2]	Encountered in extracurricular reading [2]
Cannabis	75.6 (17.7)	43.9 (28.4)	60.1 (26.5)
Cocaine	65.6 (22.2)	42.5 (27.5)	46.0 (28.4)
Heroin	60.2 (21.7)	39.5 (29.8)	41.3 (30.0)
Ketamine	61.2 (22.5)	32.2 (27.5)	41.9 (28.9)
LSD	56.1 (27.9)	16.8 (19.7)	39.8 (33.7)
Magic mushrooms	57.5 (29.7)	14.1 (19.9)	36.4 (33.0)
MDMA (ecstasy)	64.5 (23.4)	27.8 (25.5)	40.0 (31.1)

Note. All variables represent the mean (and standard deviation).

[1] Ranging from 0 (no knowledge) to 100 (excellent knowledge).

[2] Ranging from 0 (never) to 100 (very often).

For peer review only

Table 3. Multidimensional harm assessment of seven drugs (N = 132).

	Drug-specific mortality	Drug-related mortality	Drug-specific damage	Dependence	Drug-specific impairment of mental functioning	Drug-related impairment of mental functioning
Cannabis	13.3 (17.6)	38.2 (25.5)	34.9 (25.5)	48.5 (27.5)	58.4 (27.4)	48.9 (26.7)
Cocaine	70.8 (21.4)	66.4 (20.2)	73.5 (17.9)	80 (16.9)	64 (23.7)	68.4 (22.8)
Heroin	89.9 (14.3)	85.3 (16.9)	84.5 (15.7)	93.9 (11.9)	69.5 (25.5)	89.3 (13.8)
Ketamine	57.4 (27.7)	49.4 (25)	54.6 (24.8)	49.7 (25.8)	52.6 (25)	49.6 (24.9)
LSD	35.5 (28.7)	39.4 (28.1)	36.5 (28.9)	31.1 (26.5)	60.9 (26.1)	39.4 (27.3)
Magic mushrooms	28.1 (26.2)	32.3 (25.6)	29.4 (24.6)	27.2 (23.7)	55.2 (28)	36.5 (27.2)
MDMA (ecstasy)	63.5 (26.7)	53.7 (25.3)	55.9 (25.6)	48.1 (26.6)	59 (26.2)	48 (24.6)

Note. All values represent the mean (and 95% confidence interval) on a scale ranging from 0 (no harm) to 100 (severe harm).

For peer review only

Table 4. Multivariable linear regression models of the association with support for legal change ($N = 132$).

	Legal change composite scores [1]		
	Full model		
Explanatory variables (unstandardised)	Estimate	95% CI	*p*-value
Psychedelic knowledge	0.31	0.13 to 0.48	0.001
Psychedelic harm	-0.43	-0.66 to -0.21	<0.001
Course year (1 year)	2.82	0.16 to 5.48	0.038
Interest in psychiatry	-0.68	-4.01 to 2.65	0.687
Perception of pharmacological treatments	-0.01	-0.22 to 0.2	0.927
Perception of nonpharmacological treatments	-0.19	-0.38 to 0	0.051
Openness to experience	1.62	-2.4 to 5.64	0.426
Neuroticism	-0.10	-3.35 to 3.16	0.952
	Final prediction model		
Explanatory variables (unstandardised)	Estimate	95% CI	*p*-value
Psychedelic knowledge	0.31	0.14 to 0.48	<0.001
Psychedelic harm	-0.45	-0.65 to -0.24	<0.001
Course year (1 year)	3.00	0.4 to 5.59	0.024
Perception of nonpharmacological treatments	-0.18	-0.36 to 0	0.044

Abbreviations: CI, confidence interval.

[1] Ranging from 0 (strongly oppose) to 100 (strongly support).

[2] The final model retained all explanatory variables with $p < 0.05$.

For peer review only

Figure 1. Drugs ordered by their overall harm scores, showing sub-scores for each of the six harm criteria. Harm scores range from 0 (no harm) to 100 (severe harm). DSM = drug-specific mortality. DRM = drug-related mortality. DSD = drug-specific damage. DEP = dependence. DSI = drug-specific impairment of mental functioning. DRI = drug-related impairment of mental functioning. LSD = lysergic acid diethylamide.

For peer review only

References

1. Carhart-Harris RL, Goodwin GM. The Therapeutic Potential of Psychedelic Drugs: Past, Present, and Future. Neuropsychopharmacology. 2017 Oct;42(11):2105–13.

2. Nichols DE. Psychedelics. Barker EL, editor. Pharmacol Rev. 2016 Apr 1;68(2):264–355.

3. Nutt D, Erritzoe D, Carhart-Harris R. Psychedelic Psychiatry's Brave New World. Cell. 2020 Apr 2;181(1):24–8.

4. Rucker JJH, Iliff J, Nutt DJ. Psychiatry & the psychedelic drugs. Past, present & future. Neuropharmacology. 2018 Nov 1;142:200–18.

5. Nichols DE, Walter H. The History of Psychedelics in Psychiatry. Pharmacopsychiatry. 2021 Jul;54(4):151–66.

6. Nichols D, Johnson M, Nichols C. Psychedelics as Medicines: An Emerging New Paradigm. Clin Pharmacol Ther. 2017;101(2):209–19.

7. Andersen KAA, Carhart-Harris R, Nutt DJ, Erritzoe D. Therapeutic effects of classic serotonergic psychedelics: A systematic review of modern-era clinical studies. Acta Psychiatr Scand. 2021;143(2):101–18.

8. Reiff CM, Richman EE, Nemeroff CB, Carpenter LL, Widge AS, Rodriguez CI, et al. Psychedelics and Psychedelic-Assisted Psychotherapy. Am J Psychiatry. 2020 May;177(5):391–410.

9. Johnson MW, Hendricks PS, Barrett FS, Griffiths RR. Classic psychedelics: An integrative review of epidemiology, therapeutics, mystical experience, and brain network function. Pharmacol Ther. 2019 May 1;197:83–102.

10. Carhart-Harris R, Giribaldi B, Watts R, Baker-Jones M, Murphy-Beiner A, Murphy R, et al. Trial of Psilocybin versus Escitalopram for Depression. N Engl J Med. 2021 Apr 15;384(15):1402–11.

11. Raison CL, Sanacora G, Woolley J, Heinzerling K, Dunlop BW, Brown RT, et al. Single-Dose Psilocybin Treatment for Major Depressive Disorder: A Randomized Clinical Trial. JAMA. 2023 Sep 5;330(9):843–53.

12. Rotz R von, Schindowski EM, Jungwirth J, Schuldt A, Rieser NM, Zahoranszky K, et al. Single-dose psilocybin-assisted therapy in major depressive disorder: a placebo-controlled, double-blind, randomised clinical trial. eClinicalMedicine [Internet]. 2023 Feb 1 [cited 2023 Nov 29];56. Available from: https://www.thelancet.com/journals/eclinm/article/PIIS2589-5370(22)00538-7/fulltext?trk=public_post_reshare_feed-article-content

13. Goodwin GM, Aaronson ST, Alvarez O, Atli M, Bennett JC, Croal M, et al. Single-dose psilocybin for a treatment-resistant episode of major depression: Impact on patient-reported depression severity, anxiety, function, and quality of life. J Affect Disord. 2023 Apr 14;327:120–7.

For peer review only

14. Mertens LJ, Koslowski M, Betzler F, Evens R, Gilles M, Jungaberle A, et al. Methodological challenges in psychedelic drug trials: Efficacy and safety of psilocybin in treatment-resistant major depression (EPIsoDE) – Rationale and study design. Neurosci Appl. 2022 Jan 1;1:100104.

15. Mitchell JM, Bogenschutz M, Lilienstein A, Harrison C, Kleiman S, Parker-Guilbert K, et al. MDMA-assisted therapy for severe PTSD: a randomized, double-blind, placebo-controlled phase 3 study. Nat Med. 2021 Jun;27(6):1025–33.

16. Illingworth BJ, Lewis DJ, Lambarth AT, Stocking K, Duffy JM, Jelen LA, et al. A comparison of MDMA-assisted psychotherapy to non-assisted psychotherapy in treatment-resistant PTSD: A systematic review and meta-analysis. J Psychopharmacol (Oxf). 2021 May 1;35(5):501–11.

17. aan het Rot M, Zarate CA, Charney DS, Mathew SJ. Ketamine for Depression: Where Do We Go from Here? Biol Psychiatry. 2012 Oct 1;72(7):537–47.

18. Meshkat S, Haikazian S, Di Vincenzo JD, Fancy F, Johnson D, Chen-Li D, et al. Oral ketamine for depression: An updated systematic review. World J Biol Psychiatry. 2023 Aug 9;24(7):545–57.

19. Xu Y, Hackett M, Carter G, Loo C, Gálvez V, Glozier N, et al. Effects of Low-Dose and Very Low-Dose Ketamine among Patients with Major Depression: a Systematic Review and Meta-Analysis. Int J Neuropsychopharmacol. 2016 Apr 1;19(4):pyv124.

20. Aday JS, Carhart-Harris RL, Woolley JD. Emerging Challenges for Psychedelic Therapy. JAMA Psychiatry. 2023 Jun 1;80(6):533–4.

21. Barnett BS, Beaussant Y, King F, Doblin R. Psychedelic Knowledge and Opinions in Psychiatrists at Two Professional Conferences: An Exploratory Survey. J Psychoactive Drugs. 2022 May 27;54(3):269–77.

22. Barnett BS, Siu WO, Pope HGJ. A Survey of American Psychiatrists' Attitudes Toward Classic Hallucinogens. J Nerv Ment Dis. 2018 Jun;206(6):476.

23. Page LA, Rehman A, Syed H, Forcer K, Campbell G. The Readiness of Psychiatrists to Implement Psychedelic-Assisted Psychotherapy. Front Psychiatry [Internet]. 2021 [cited 2023 Nov 29];12. Available from: https://www.frontiersin.org/articles/10.3389/fpsyt.2021.743599

24. Davis AK, Agin-Liebes G, España M, Pilecki B, Luoma J. Attitudes and Beliefs about the Therapeutic Use of Psychedelic Drugs among Psychologists in the United States. J Psychoactive Drugs. 2022 Aug 8;54(4):309–18.

25. Hearn BG, Brubaker MD, Richardson G. Counselors' attitudes toward psychedelics and their use in therapy. J Couns Dev. 2022;100(4):364–73.

26. Wang K, Sun Y, Nava B, Sampiere L, Jacobs RJ. Predictors of Medical Students' Perceptions of Psilocybin-Assisted Therapy for Use in Medical Practice. Cureus. 2023;15(4):e37450.

For peer review only - http://bmjopen.bmj.com/site/about/guidelines.xhtml

27. Li I, Fong R, Hagen M, Tabaac B. Medical student attitudes and perceptions of psychedelic-assisted therapies. Front Psychiatry. 2023 Jun 27;14:1190507.

28. Pagán AF, Lex C, Soares JC, Meyer TD. Medical Students' Attitudes and Beliefs Toward Psilocybin: Does Terminology and Personal Experience with Psychedelics Matter? Psychedelic Med. 2023 Sep;1(3):130–8.

29. Nutt DJ, King LA, Phillips LD. Drug harms in the UK: a multicriteria decision analysis. The Lancet. 2010 Nov 6;376(9752):1558–65.

30. Cheeta S, Halil A, Kenny M, Sheehan E, Zamyadi R, Williams AL, et al. Does perception of drug-related harm change with age? A cross-sectional online survey of young and older people. BMJ Open. 2018 Nov 1;8(11):e021109.

31. Prohibition Partners. PSYCH: The Psychedelics as Medicine Report Second Edition. 2020.

32. Rammstedt B, John OP. Measuring personality in one minute or less: A 10-item short version of the Big Five Inventory in English and German. J Res Personal. 2007 Feb 1;41(1):203–12.

33. John OP, Srivastava S. The Big Five Trait taxonomy: History, measurement, and theoretical perspectives. In: Handbook of personality: Theory and research, 2nd ed. New York, NY, US: Guilford Press; 1999. p. 102–38.

For peer review only

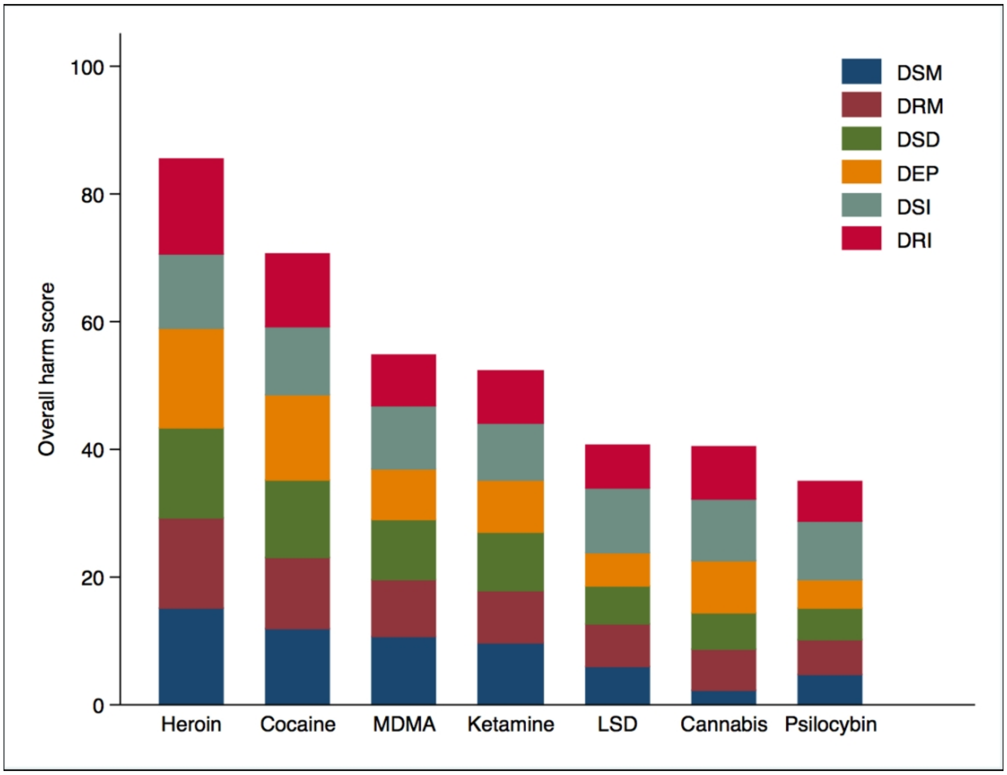

Figure 1. Drugs ordered by their overall harm scores, showing sub-scores for each of the six harm criteria. Harm scores range from 0 (no harm) to 100 (severe harm). DSM = drug-specific mortality. DRM = drug-related mortality. DSD = drug-specific damage. DEP = dependence. DSI = drug-specific impairment of mental functioning. DRI = drug-related impairment of mental functioning. LSD = lysergic acid diethylamide.

222x170mm (144 x 144 DPI)

Reporting checklist for cross sectional study.

Based on the STROBE cross sectional guidelines.

Instructions to authors

Complete this checklist by entering the page numbers from your manuscript where readers will find each of the items listed below.

Your article may not currently address all the items on the checklist. Please modify your text to include the missing information. If you are certain that an item does not apply, please write "n/a" and provide a short explanation.

Upload your completed checklist as an extra file when you submit to a journal.

In your methods section, say that you used the STROBE cross sectionalreporting guidelines, and cite them as:

von Elm E, Altman DG, Egger M, Pocock SJ, Gotzsche PC, Vandenbroucke JP. The Strengthening the Reporting of Observational Studies in Epidemiology (STROBE) Statement: guidelines for reporting observational studies.

		Reporting Item	Page Number
**Title and abstract**			
Title	#1a	Indicate the study's design with a commonly used term in the title or the abstract	1
Abstract	#1b	Provide in the abstract an informative and balanced summary of what was done and what was found	2
**Introduction**			
Background / rationale	#2	Explain the scientific background and rationale for the investigation being reported	4
Objectives	#3	State specific objectives, including any prespecified hypotheses	6
**Methods**			
Study design	#4	Present key elements of study design early in the paper	7
Setting	#5	Describe the setting, locations, and relevant dates, including periods of	7

For peer review only

		recruitment, exposure, follow-up, and data collection	
Eligibility criteria	#6a	Give the eligibility criteria, and the sources and methods of selection of participants.	7
	#7	Clearly define all outcomes, exposures, predictors, potential confounders, and effect modifiers. Give diagnostic criteria, if applicable	8
Data sources / measurement	#8	For each variable of interest give sources of data and details of methods of assessment (measurement). Describe comparability of assessment methods if there is more than one group. Give information separately for for exposed and unexposed groups if applicable.	8
Bias	#9	Describe any efforts to address potential sources of bias	7
Study size	#10	Explain how the study size was arrived at	8
Quantitative variables	#11	Explain how quantitative variables were handled in the analyses. If applicable, describe which groupings were chosen, and why	8
Statistical methods	#12a	Describe all statistical methods, including those used to control for confounding	12
Statistical methods	#12b	Describe any methods used to examine subgroups and interactions	n/a
Statistical methods	#12c	Explain how missing data were addressed	n/a
Statistical methods	#12d	If applicable, describe analytical methods taking account of sampling strategy	n/a
Statistical methods	#12e	Describe any sensitivity analyses	n/a
**Results**			
Participants	#13a	Report numbers of individuals at each stage of study—eg numbers potentially eligible, examined for eligibility, confirmed eligible, included in the study, completing follow-up, and analysed. Give information separately for for exposed and unexposed groups if applicable.	12
Participants	#13b	Give reasons for non-participation at each stage	n/a
Participants	#13c	Consider use of a flow diagram	n/a

For peer review only

Descriptive data	#14a	Give characteristics of study participants (eg demographic, clinical, social) and information on exposures and potential confounders. Give information separately for exposed and unexposed groups if applicable.	12
Descriptive data	#14b	Indicate number of participants with missing data for each variable of interest	n/a
Outcome data	#15	Report numbers of outcome events or summary measures. Give information separately for exposed and unexposed groups if applicable.	12
Main results	#16a	Give unadjusted estimates and, if applicable, confounder-adjusted estimates and their precision (eg, 95% confidence interval). Make clear which confounders were adjusted for and why they were included	13
Main results	#16b	Report category boundaries when continuous variables were categorized	n/a
Main results	#16c	If relevant, consider translating estimates of relative risk into absolute risk for a meaningful time period	n/a
Other analyses	#17	Report other analyses done—e.g., analyses of subgroups and interactions, and sensitivity analyses	n/a
**Discussion**			
Key results	#18	Summarise key results with reference to study objectives	14
Limitations	#19	Discuss limitations of the study, taking into account sources of potential bias or imprecision. Discuss both direction and magnitude of any potential bias.	16
Interpretation	#20	Give a cautious overall interpretation considering objectives, limitations, multiplicity of analyses, results from similar studies, and other relevant evidence.	17
Generalisability	#21	Discuss the generalisability (external validity) of the study results	16
**Other Information**			
Funding	#22	Give the source of funding and the role of the funders for the present study and, if applicable, for the original study on which the present article is based	1

The STROBE checklist is distributed under the terms of the Creative Commons Attribution License CC-BY.
This checklist was completed on 21. December 2023 using https://www.goodreports.org/, a tool made by the EQUATOR Network in collaboration with Penelope.ai

For peer review only

BMJ Open

UK medical students’ self-reported knowledge and harm assessment of psychedelics and their application in clinical research: a cross-sectional study

Journal:	*BMJ Open*
Manuscript ID	bmjopen-2023-083595.R1
Article Type:	Original research
Date Submitted by the Author:	15-Feb-2024
Complete List of Authors:	Song-Smith, Charlie; University College London, UCL Medical School<br>Jacobs, Edward; University of Oxford, Department of Psychiatry; University of Oxford, Wellcome Centre for Ethics and Humanities<br>Rucker, James; King's College London, The Department of Psychological Medicine; South London and Maudsley NHS Foundation Trust<br>Saint, Matthew; University of Exeter Medical School<br>Cooke, James; Innerspace Institute<br>Schlosser, Marco; UCL, Division of Psychiatry; Institut für Psychotherapie Potsdam
**Primary Subject Heading**:	Medical education and training
Secondary Subject Heading:	Medical education and training
Keywords:	MEDICAL EDUCATION & TRAINING, Cross-Sectional Studies, PSYCHIATRY

SCHOLARONE™
Manuscripts

I, the Submitting Author has the right to grant and does grant on behalf of all authors of the Work (as defined in the below author licence), an exclusive licence and/or a non-exclusive licence for contributions from authors who are: i) UK Crown employees; ii) where BMJ has agreed a CC-BY licence shall apply, and/or iii) in accordance with the terms applicable for US Federal Government officers or employees acting as part of their official duties; on a worldwide, perpetual, irrevocable, royalty-free basis to BMJ Publishing Group Ltd ("BMJ") its licensees and where the relevant Journal is co-owned by BMJ to the co-owners of the Journal, to publish the Work in this journal and any other BMJ products and to exploit all rights, as set out in our licence.

The Submitting Author accepts and understands that any supply made under these terms is made by BMJ to the Submitting Author unless you are acting as an employee on behalf of your employer or a postgraduate student of an affiliated institution which is paying any applicable article publishing charge ("APC") for Open Access articles. Where the Submitting Author wishes to make the Work available on an Open Access basis (and intends to pay the relevant APC), the terms of reuse of such Open Access shall be governed by a Creative Commons licence – details of these licences and which Creative Commons licence will apply to this Work are set out in our licence referred to above.

Other than as permitted in any relevant BMJ Author's Self Archiving Policies, I confirm this Work has not been accepted for publication elsewhere, is not being considered for publication elsewhere and does not duplicate material already published. I confirm all authors consent to publication of this Work and authorise the granting of this licence.

For peer review only

UK medical students' self-reported knowledge and harm assessment of psychedelics and their application in clinical research: a cross-sectional study

Charlie Song-Smith[a, 1], Edward Jacobs[b, c], James Rucker[d, e], Matthew Saint[f], James Cooke[g], & Marco Schlosser[h, i, 1]

[a] UCL Medical School, University College London, London, United Kingdom
[b] Department of Psychiatry, University of Oxford, Oxford, United Kingdom
[c] Wellcome Centre for Ethics and Humanities, University of Oxford, Oxford, United Kingdom
[d] The Department of Psychological Medicine, Institute of Psychiatry, Psychology & Neuroscience, King's College London, London, United Kingdom
[e] South London & Maudsley NHS Foundation Trust, Monks Orchard Road, Beckenham, Kent, United Kingdom
[f] University of Exeter Medical School, Exeter, UK
[g] Innerspace Institute, Portugal
[h] Division of Psychiatry, Faculty of Brain Sciences, University College London, London, United Kingdom
[i] Institut für Psychotherapie Potsdam, Potsdam, Germany

Marco Schlosser and James Cooke share last authorship.

[1] Corresponding authors: Charlie Song-Smith & Marco Schlosser

Charlie Song-Smith,
Email: charlie.song-smith.19@ucl.ac.uk
UCL Medical School
University College London
London, United Kingdom

Marco Schlosser
Email: marco.schlosser@ucl.ac.uk
Division of Psychiatry
University College London
London, United Kingdom

For peer review only

For peer review only - http://bmjopen.bmj.com/site/about/guidelines.xhtml

Abstract

Objective: To capture UK medical students' self-reported knowledge and harm assessment of psychedelics and to explore the factors associated with support for changing the legal status of psychedelics to facilitate further clinical research.

Design: Cross-sectional, anonymous online survey of UK medical students using a non-random sampling method.

Setting: UK medical schools recognised by the General Medical Council.

Participants: 132 medical students who had spent an average of 3.8 years (SD=1.4; range: 1 to 6) in medical school.

Results: Most students (83%) reported that they were aware of psychedelic research and only four participants (3%) said that they were not interested in learning more about this type of research. Although medical students' harm assessment of psychedelics closely aligned with that of experts, only 17% of students felt well-educated on psychedelic research. Teachings on psychedelics were only rarely encountered in their curriculum (psilocybin: 14.1 [SD=19.9], scale: 0 [never] to 100 [very often]). Time spent at medical schools was not associated with more knowledge about psychedelics ($r=0.12$, $p=0.129$). On average, this sample of medical students showed strong support for changing the legal status of psychedelics to facilitate further research into their potential clinical applications (psilocybin: 80.2 [SD=24.8], scale: 0 [strongly oppose] to 100 [strongly support]). Regression modelling indicated that greater knowledge of psychedelics ($p<0.001$), lower estimated harm scores ($p<0.001$), more time spent in medical school ($p=0.024$), and lower perceived effectiveness of nonpharmacological mental health treatments ($p=0.044$) were associated with greater support for legal status change.

Conclusions: Our findings reveal a significant interest among UK medical students to learn more about psychedelic research and a strong support for further psychedelic research. Future studies are needed to examine how medical education could be refined to adequately prepare medical students for a changing healthcare landscape in which psychedelic-assisted therapy could soon be implemented in clinical practice.

Keywords: Medical students, Psychedelics, Knowledge, Harm assessment, Medical education

Word count: 4618

For peer review only - http://bmjopen.bmj.com/site/about/guidelines.xhtml

Strengths and limitations of this study

- This study presents the first survey of UK medical students' attitudes towards psychedelics and their application in clinical research.
- The wide-ranging assessment included medical students' self-reported knowledge of psychedelics, a multidimensional harm assessment of psychedelics, level of support for changing the legal status of psychedelics to facilitate further research into their potential clinical applications, and students' perceptions of medical education on psychedelics and psychedelic research.
- The cross-sectional nature of our data prevented us from drawing causal conclusions about potential changes in students' attitudes and knowledge over time.
- The limited range of demographic variables did not allow for an in-depth characterisation of this sample and important predictors of attitudes might have been missed. Non-random sampling and selection bias could have impacted the generalisability of our findings as students already interested in drugs might have been more likely to participate.

For peer review only

For peer review only - http://bmjopen.bmj.com/site/about/guidelines.xhtml

Introduction

Since the early 1990s, there has been a steady resurgence of research involving psychedelics such as lysergic acid diethylamide (LSD), psilocybin, and N, N-Dimethyltryptamine (DMT) (1–3). After a short-lived period of medical use of psychedelics by clinicians and researchers in the middle of the 20th century, psychedelic research was effectively ended by prohibitive legislature and a lack of governmental research funding (4). The clinical trials conducted prior to these legal sanctions suggested that psychedelics – which were frequently used in combination with or as an aid to psychotherapy – could positively impact a range of disorders, particularly mood disorders and alcohol dependence (5,6). These early findings have been corroborated by modern-era clinical trials, which support the therapeutic effects of psychedelics and psychedelic-assisted psychotherapy in some patient groups (for reviews see e.g, 7–10). The largest published and ongoing psychedelic trials investigate the effects of psilocybin in combination with psychological support for the treatment of major depressive disorder (e.g, 11–15). Clinical investigations into the therapeutic efficacy of related drugs, such as 3,4-Methylenedioxymethamphetamine (MDMA) for post-traumatic stress disorder (PTSD) (16,17) and ketamine for depression (18–20), have also advanced substantially. In the context of the present study, the term “psychedelic” will encompass the classic psychedelics (e.g., psilocybin, LSD) as well as MDMA and ketamine.

If current developments continue unhindered, some psychedelics and forms of psychedelic-assisted therapy will likely receive regulatory approval and thus transition from legally restricted use in research studies to integration in clinical practice (but see 21)). Several companies are undertaking efforts to commercialize psychedelics as medical treatments (see 22). The extent to which psychedelics will be clinically implemented and disseminated will also depend on medical professionals’ knowledge and attitudes towards

For peer review only - http://bmjopen.bmj.com/site/about/guidelines.xhtml

these drugs. Despite the looming possibility of regulatory approval, research on medical professionals' opinions surrounding psychedelic and their use in clinical practice is in its infancy. A recent survey indicated that US psychiatrists (n=106) largely believed in the therapeutic potential of psychedelics and were strongly in favour of federal funding to facilitate further research with these substances (23). Variables associated with a favourable assessment of psychedelics included greater psychedelic knowledge, fewer concerns about the drugs' addictive potential, and working in research. A 2016 survey study led by the same author had also suggested that psychiatrists (n=324) were supportive of further psychedelic research on mental health disorders, but the sample also tended to view these drugs as potentially dangerous and agreed with their restrictive scheduling for recreational use (24). A 7-year follow-up survey (n=131) found that optimism among psychiatrists regarding the clinical potential of psychedelics had increased while concerns about risk had decreased; 50% of psychiatrists indicated moderate or strong intentions to integrate psychedelic therapy into their clinical practice (25). A pilot study (n=83) of psychiatrists working in the UK's National Health Service (NHS) showed that most of them (77%) believed that controlled or therapeutic use of psychedelics should play a role in society (26). Notably, psychiatrists of all grades reported significant training needs and lacking preparedness or confidence to deliver psychedelic-assisted psychotherapy should it become available. Overall, most research conducted in this nascent field converges on the conclusion that a majority of psychiatrists and other mental health professionals – including psychologists and counsellors (27,28) – are supportive of further clinical research with psychedelics.

Given that tomorrow's psychiatrists are today's medical students, it is pertinent to consider medical students' knowledge and attitudes towards psychedelics. To date, only three studies on this topic have been published (29–31). In a sample of 213 US medical students, 83% and 52% of respondents believed that psilocybin should be legalised for recreational use

For peer review only

and medical use, respectively (29). Students with greater self-reported knowledge of psilocybin and fewer concerns about potential negative effects reported more positive overall perceptions regarding the medical use of psilocybin. Another survey study of US medical students (n=132) suggested that almost all respondents (95%) believed that further research examining psychedelics' therapeutic potential is warranted (30). Overall, medical students held positive attitudes toward psychedelics, but only 41% reported that they felt knowledgeable about psychedelics. None of the demographic variables, including age, sex, planned specialty (i.e., psychiatry, internal medicine etc.), and level of training, were associated with greater positive attitudes towards psychedelics. Lastly, another survey of US medical students (n=295) indicated that more favourable attitudes towards psychedelics were associated with a greater willingness to recommend psychedelic-assisted therapy to patients should it receive FDA approval (31). The present study aimed to further expand knowledge in this area by sampling the views and attitudes of medical students in the United Kingdom.

Using an anonymous online survey, we aimed to capture UK medical students' self-reported knowledge of psychedelics and their application in clinical research. We also aimed to compare medical students' harm assessment of psychoactive drugs with that of drug experts. We aimed to examine to which extent participant characteristics were associated with support for changing the legal status of psychedelics to facilitate further research. We expected more time spent in medical school to be associated with higher psychedelic knowledge and lower psychedelic harm composite scores. Regarding support for changing the legal status of psychedelics to facilitate further research, we expected students more knowledgeable of psychedelics to be more supportive of their legal status change; students assigning lower harm to psychedelics to be more supportive; more seasoned students to be more supportive; more psychiatrically interested students to be more supportive; students favourably assessing pharmacological and nonpharmacological to be more and less

For peer review only - http://bmjopen.bmj.com/site/about/guidelines.xhtml

supportive, respectively; students more open to new experiences to be more supportive; and students with higher levels of neuroticism to be less supportive.

Methods

Procedures and survey design

This study used an anonymous online survey delivered via the Qualtrics web platform to collect cross-sectional data without recording participants' IP addresses. A short text asking individuals whether they would like to support research on medical students' knowledge and perceptions of common "recreational" drugs and their clinical uses was shared on UK university medical society mailing lists and bulletins, and social media platforms (LinkedIn, Twitter). To reduce self-selection bias, the study was advertised as a "Drugs and Mental Health" survey, rather than a survey about psychedelics. Interested individuals could follow a link to the study information sheet, which described the aims of the survey, the anonymous data collection, and the eligibility criteria (i.e. at least 18 years old, good understanding of the English language, currently enrolled in a UK medical programme accredited by the General Medical Council). Before starting the survey, individuals were required to indicate their informed consent by agreeing to the following statement: "I have read and understand the explanations, I am at least 18 years old and have a good understanding of the English language, and I voluntarily consent to participate in this study". The survey took approximately 10 minutes to complete and was accessible between October 2021 and March 2022. No financial or other incentives were offered for participation. This study received ethical approval from University College London's Research Ethics Committee (Project ID: 14309/007) and was performed in accordance with the 1964 Declaration of Helsinki and its later amendments. All data was collected and processed in accordance with the 2018 UK General Data Protection Regulation. The full survey can be found in the Supplementary Material.

For peer review only - http://bmjopen.bmj.com/site/about/guidelines.xhtml

Participants

A total of 136 individuals consented to participate and completed the survey. Incomplete responses were automatically deleted by Qualtrics after one month. In the service of survey quality control, participants who took less than five minutes to complete the survey were excluded, because this presents the time it approximately took to read the survey questions at a fast pace, but without responding to any questions (i.e., taking less than five minutes was judged to be indicative of participants not properly reading the survey questions or response options). Four participants were thus excluded. The total sample consisted of medical 132 students without missing data.

Measures

To ensure anonymity, we collected only minimal demographic information including course year (i.e., years since starting training), participation in intercalated BSc or MBPhD programme, previous completion of university degree, and interest in pursuing a career in psychiatry.

To capture medical students' self-reported knowledge and the amount of education they had received on seven drugs (i.e, cannabis, cocaine, heroin, ketamine, LSD, magic mushrooms/psilocybin, ecstasy/MDMA), we used the following three statements:

1. Self-reported knowledge: "Firstly, please indicate how well educated/knowledgeable you feel about these drugs. For example, this would include knowledge of pharmacology, physiology, subjective effects and adverse consequences of the drug."
2. Encountered in curriculum: "How often have you come across the drugs listed above in your teaching, including lectures, small group work and problem-based or self-directed learning?"
3. Encountered in extracurricular reading: "How often have you come across these drugs in your own reading/research?"

For peer review only

The first statement used a sliding visual analogue scale (VAS) ranging from 0 ('no knowledge') to 100 ('excellent knowledge'), whilst the latter two questions used a VAS from 0 ('never') to 100 ('very often'). Commonly used non-psychedelic drugs were included to allow comparison of relative perceived harms, as well as to cover the fact that this was primarily a psychedelic survey. A psychedelic knowledge composite was computed by averaging the knowledge scores of LSD, psilocybin, MDMA, and ketamine (i.e., four scale scores were combined), with higher composite scores indicating greater self-reported knowledge.

To measure medical students' perceptions of harms of the same seven drugs, the following six questions with a VAS from 0 (no harm) to 100 (severe harm) were used:

4. Drug-specific mortality: "How great is the potential of the drug to cause death by overdose (i.e., the intrinsic lethality)?"
5. Drug-related mortality: "To what extent is life shortened by the use of the drug, excluding deaths from overdose? For example, this could include deaths due to road traffic accidents, lung cancers, HIV and suicide."
6. Drug-specific damage: "How great is the damage to physical health (short of death) caused by the drug? For example, this could include liver damage, other organ damage, seizures, strokes."
7. Dependence: "To what extent does the drug cause addiction/dependence, i.e., the urge to continue to use despite adverse consequences?"
8. Drug-specific impairment of mental functioning: "To what extent does the drug cause acute mental health problems when taken?"
9. Drug-related impairment of mental functioning: "To what extent does the drug cause problems in the user's work or social life, leading to mental health problems (excluding addiction)?"

For peer review only - http://bmjopen.bmj.com/site/about/guidelines.xhtml

A psychedelic harm composite score was computed by averaging the harm scores of LSD, psilocybin, MDMA, and ketamine across the six harm assessment categories (i.e., 24 scale scores were combined), with higher composite scores indicating higher levels of harm. The harm-related questions were adopted from a cross-sectional survey (32) that utilised the 16 harm criteria introduced by a multidisciplinary panel of drug experts in a seminal paper on relative drug harms on behalf of the in the UK's Independent Scientific Committee on Drugs (33). Using a brief survey to minimise attrition rate, we adopted only six of the 16 criteria covering physical harm (drug-specific mortality, drug-related mortality, drug-specific damage) and psychological harm (dependence, drug-specific impairment of mental functioning, drug-related impairment of mental functioning). Detailed information about these harm criteria can be found Nutt et al. (29).

To capture medical students' attitudes towards mental health treatments and research on psychedelics, we used the following questions and scales:

10. Perception of effectiveness of pharmacological (e.g., selective-serotonin reuptake inhibitors [SSRIs] and nonpharmacological treatments (e.g., psychotherapy): "Thinking about mental health conditions such as anxiety disorders, depression and addiction, how effective would you consider the following general treatment approaches for these conditions?" (VAS from 0 [not effective at all] to 100 [extremely effective])
11. Awareness of and interest in psychedelic research (adapted from the Psychedelic as Medicine Report, (34)): "Consider the following statement: 'Drugs such as psilocybin (the active ingredient in magic mushrooms) and MDMA (the active ingredient in ecstasy) have recently been investigated as potential treatments for mental health conditions such as depression, PTSD, and addiction.' Which of the following best describes your own awareness of this research area?" (5-point

For peer review only

nominal scale: I am aware of this, and am well educated on the topic, I am aware of this, and curious to know more; I am aware of this, but it doesn't interest me; I am not aware of this, but am curious to know more; I am not aware of this, and it doesn't interest me).

12. Attitude towards changing legal status of psilocybin and MDMA: “Consider the following statement: ‘Drugs that are considered to have no medical value and a high risk of misuse or harm are listed in Schedule 1 of the UK Misuse of Drugs Regulations (2001); these currently include MDMA and psilocybin. Research involving schedule 1 drugs is associated with significantly increased costs, duration, and difficulty.’ Given your current knowledge, to what extent would you support or oppose changing the legal status of these drugs to facilitate further research into their potential medical uses (while keeping restrictions on recreational use as they are)?” (VAS from 0 [strongly oppose] to 100 [strongly support])

A ‘legal change’ composite variable was created by averaging the psilocybin and MDMA legal change scores, with higher scores indicating greater support for changing the legal status to facilitate further research.

Another drug-related question asked medical students to rate, using a VAS from 0 (not important) to 100 (very important), the importance of the following factors in influencing their perception of the drugs included in this survey: my own and my friends’ personal experiences; teaching as part of my course; media reporting; reading scientific papers/articles; a drug's legal class; advice from parents or other authority figures; general internet searches and online information.

To measure personality traits, the short version of the Big Five Inventory (BFI) was used (35). This 10-item scale uses a 5-point Likert scale ranging from 1 (disagree strongly) to 5 (agree strongly) to capture openness to experience, conscientiousness, extraversion,

For peer review only - http://bmjopen.bmj.com/site/about/guidelines.xhtml

agreeableness, and neuroticism (36). Each personality trait is captured by two items and total scores for each trait are computed by averaging the respective items scores. Higher subscale scores relate to higher trait levels. The 10-item version, despite being significantly shorter than the standard 44-item Big Five Inventory on which it is based, has retained good levels of reliability and validity (35).

Data analysis

Sample size

Given the lack of studies that have utilised regression modelling to investigate the potential predictors of medical students' support for changing the legal status of psychedelics, we were not able to conduct an *a priori* power analysis based on a published effect size of this relationship. Instead, we aimed to recruit as many UK medical students as possible over a 6-month period.

Associations of drug-related measures with course year

The relationship between course year and drug-related measures was assessed using Pearson's correlation coefficients.

Regression analysis

We built the statistical models within a risk prediction framework using a stepwise backward elimination approach. The full mode considered the legal change composite score as the continuous outcome. Given the lack of research on medical students' attitudes towards psychedelic research that could guide the selection of potential predictors, we decided to include the following variables as continuous explanatory variables: psychedelic knowledge composite scores, psychedelic harm composite scores, course year, interest in psychiatry, perception of effectiveness of mental health treatments, openness to experience, and neuroticism. The explanatory variable associated with the highest *p*-value above 0.05 was then deleted. The model was then refitted without the deleted explanatory variable and the

For peer review only

estimates and their associated *p*-values recomputed. The final prediction model thus retained explanatory variables if they were associated with $p < 0.05$. This threshold for inclusion was chosen to ensure model stability and reduce overfitting. Stata/MP version 16.0 and R version 4.0.2 were used for all analyses.

Patient and public involvement statement

Patients or the public were not involved in the development, design, conduct, reporting, or dissemination plans of our research.

Results

Descriptive statistics

Participant characteristics for the total sample of 132 medical students are displayed in Table 1. We used Qualtrics survey settings that deleted incomplete responses after one month. A response rate could, therefore, not be computed. Descriptive statistics of self-reported knowledge and the extent to which medical students had encountered a discussion of the listed drugs (i.e., cannabis, cocaine, heroin, ketamine, LSD, psilocybin, MDMA) in their curriculum or during extracurricular reading and research are displayed in Table 2. Descriptive statistics on medical students' assessment of the harm caused by the listed drugs are reported in Table 3 and visually depicted in Figure 1.

The mean rating (on a scale from 0 [strongly oppose] to 100 [strongly support]) for the change in legal status for psilocybin was 80.2 (SD = 24.8) and for MDMA was 74.3 (SD = 27.7). Both distributions were left-skewed (i.e., more values are on the right side of the mean) with 59 (44.7%) and 50 (37.9%) medical students having a score of 100 for psilocybin and MDMA, respectively. The legal change composite score showed a similar divergence from normality (skewness: -0.98, kurtosis: 3.02).

The other two composite scores did not markedly diverge from normality as indicated by estimates of skewness (psychedelic knowledge: -0.17; psychedelic harm: -0.13) and

For peer review only - http://bmjopen.bmj.com/site/about/guidelines.xhtml

kurtosis (psychedelic knowledge: 2.30; psychedelic harm: 2.34) and visual inspection of the histograms.

Associations of drug-related measures with course year

More course years were associated with lower psychedelic harm composite scores (Pearson's correlation coefficient r = -0.22, 95% CI: -0.05 to -0.38, p = 0.012). In contrast to our expectations, number of course years were not correlated with higher psychedelic knowledge composite scores (r = 0.12, 95% CI: -0.04 to 0.30, p = 0.129).

Regression analysis

Table 4 reports the results from the full model and the final prediction model. Using a stepwise backward elimination approach with significance threshold of $p = 0.05$, explanatory variables were removed in the following order: neuroticism, perception of pharmacological treatments, interest in psychiatry, and, in the last step, openness to experience. The final prediction model for legal change composite scores thus retained psychedelic knowledge composite scores, psychedelic harm composite scores, course year, and perception of the effectiveness of nonpharmacological treatments (all unstandardised estimates are displayed in Table 4). In other words, in the final regression model, greater psychedelic knowledge and more course years were associated with greater support for legal change, whereas greater psychedelic harm scores and a more favourable perception of the effectiveness of nonpharmacological treatments were associated with weaker support for legal change of psychedelics. Assumptions of linear regression – namely, independence, normality (all Ws of Shapiro Wilk test for normal data associated with p>0.123), and homoscedasticity of residuals, and linearity – were judged to be adequately met for all regression models; no notable level of multicollinearity was detected.

Discussion

For peer review only

The primary aim of this cross-sectional study was to capture UK medical students' self-reported knowledge and harm assessment of psychedelics and the degree to which they would support changing the legal status of psychedelic substances, particularly psilocybin and MDMA, to facilitate further research into their potential medical uses without changing restrictions on recreational use. Results indicated that 83% of medical students were aware of psychedelic research and only four participants said that they were not interested in learning more about this type of research. Medical students evaluated their own experience and that of their friends as the most important influence on their perception of drugs in general. Another important influence was their study of scientific articles, whereas media reporting and a drug's legal class had the least influence on their perception of drugs.

UK medical students were highly supportive of changing the legal status of psilocybin and MDMA, with 45% and 38% expressing the strongest possible support (i.e., a score of 100 on a scale from 0 [strongly oppose] to 100 [strongly support]) for psilocybin and MDMA, respectively. Relatedly, we aimed to explore the variables that would predict the extent to which medical students were in support of changing the legal status. Regression modelling suggested that students who reported greater knowledge of psychedelics, assigned lower harm scores to psychedelics, had spent more time spent in medical education, and assigned lower effectiveness scores to nonpharmacological mental health treatments showed a greater support for legal status change. The contribution of these variables was in line with our prediction. However, diverging from our expectation was the absence of a relationship between support for legal change and an interest in psychiatry, the perceived effectiveness of pharmacological treatments, and the personality traits of openness to experience and neuroticism.

Although time spent in medical school itself was associated with lower harm ratings of psychedelics, it was not associated with greater self-reported knowledge of psychedelics,

For peer review only - http://bmjopen.bmj.com/site/about/guidelines.xhtml

suggesting that medical degree curricula do not contain much teaching about psychedelics. This explanation also aligns with the low reported frequency with which medical students encountered the classic psychedelics, psilocybin and LSD, in their curriculum. An alternative explanation, for instance, that medical students attracted to a survey on drugs and mental health tend to have greater knowledge of psychedelics (resulting in ceiling effects), was not reflected in the data, which showed self-reported psychedelic knowledge to be approximately normally distributed. In this context, it is important to consider that self-treatment with psychedelics is increasing (37,38) and that harm reduction opportunities for psychedelic use among patients could be potentially missed due to lack of knowledge about psychedelics among healthcare providers. Specifically, patients are afraid to discuss psychedelic use with providers due to concerns about stigma and the assumption that their providers' knowledge base is low (39). For example, providers could encourage patients with bipolar or psychotic disorders to avoid psychedelics and inform them about potentially adverse medication interactions such as seizures from combining psychedelics with lithium (40).

Medical students' multidimensional harm assessment of seven drugs resulted in an order of assigned overall harm scores that closely matched the order that was assigned by drug expert in a widely-cited harm assessment report conducted on behalf of the UK's Independent Scientific Committee on Drugs (33). In alignment with the expert rating, medical students assigned the highest overall harm score to heroin and cocaine and the lowest to psilocybin. Differences emerged in relation to the rank order of cannabis, which experts rated as substantially more harmful than medical students. In contrast, medical students perceived MDMA to be the third most harmful substance amongst the list of seven drugs assessed in the present study, whereas experts judged the harm of MDMA to be similar to that of LSD and psilocybin.

Limitations

For peer review only - http://bmjopen.bmj.com/site/about/guidelines.xhtml

This study has important limitations that need to be considered when interpreting our results. First, the cross-sectional nature of our data prevents us from drawing causal conclusions about the association between support for changing the legal status of psychedelics and the potential predictors we have considered. Future research with larger sample sizes, more detailed demographic information, objective assessments of students' knowledge, and longitudinal follow-ups are needed to further understand how medical education shapes students' perception of psychedelics and the factors that influence the degree to which students' views align with empirical evidence and expert opinion. Second, some of the measures we applied to capture medical students' knowledge and attitudes towards psychedelics were tailor-made for this survey and not previously validated, which needs to be considered when interpreting the results. Our harm assessment was adapted from Nutt et al. (33), but we used only six of the 16 harm criteria these researchers proposed; and none of our harm criteria captured harm to others. We also did not include an assessment of other commonly used drugs, such as alcohol and tobacco, preventing us from drawing potentially insightful comparisons. The brevity of our assessment was to keep attrition rates as low as possible. We did not capture medical students' personal psychedelic use, which may be associated with attitudes towards psychedelics (see 41). Future research would benefit from a more in-depth and comprehensive assessment of UK medical students' views and attitudes surrounding psychedelics and their application in clinical research. Third, we relied on self-report data gathered via an anonymous online survey, which automatically deleted incomplete responses after one month, thus not allowing us to capture a response rate; we did not conduct an objective assessment of knowledge about psychedelics (e.g., a multiple choice test) and, therefore, cannot assess the validity and reliability of students' self-report. Lastly, generalisability of our study may be limited due to the present sample's high awareness of and interest in psychedelic research. Despite efforts to minimise selection bias

For peer review only

by avoiding the term “psychedelic” during recruitment, it is likely that the students who completed the survey were not representative of the general UK medical student population. The generalisability of our findings might also be impeded by the heterogeneity of the sample in relation to length of medical training. Overall, given the lack of empirical and theoretical work in this area, our hypotheses and statistical approaches should be viewed as primarily exploratory and our findings as preliminary (our data are openly accessible [42]).

Conclusion

This is the first study to explore UK medical students’ self-reported knowledge and perceptions of psychedelics. Medical students showed strong support for changing the legal status of psychedelics to facilitate further research into their potential clinical applications. In general, medical students only rarely encountered teachings on psychedelics in their medical education. Whilst only a small percentage of medical students felt well-educated on psychedelic research, a large majority were interested to learn more, likely reflecting the re-emergence of clinical interest in this area.

For peer review only

For peer review only - http://bmjopen.bmj.com/site/about/guidelines.xhtml

Table 1. Demographics and participant characteristics (*N* = 132).

Variable	Mean (SD) or *n* (%)
Course years (i.e., time spent in medical education)	3.8 (1.4), range: 1 to 6
First year	6 (4.6%)
Second year	14 (10.6%)
Third year	40 (30.3%)
Fourth year	32 (24.2%)
Fifth year	17 (12.9%)
Sixth year	23 (17.4%)
Enrolled in or completed an intercalated degree	62 (47.0%)
Attained a previous university degree	29 (22.0%)
Interest in psychiatry	
I am confident I will pursue a career in psychiatry.	8 (6.1%)
I consider psychiatry one of my more likely career paths.	35 (26.5%)
I have not ruled out psychiatry, but it is not one of my more likely career paths.	39 (29.6%)
I consider psychiatry one of my less likely career paths.	35 (26.5%)
I am confident I will not pursue a career in psychiatry.	15 (11.4%)
Influence on perceptions of drugs (range: 0 [not important] to 100 [very important])	
My own and my friends' personal experiences	77 (20)
Teaching as part of my course	44 (29)
Media reporting	39 (26)
Reading scientific papers/articles	65 (28)
A drug's legal class	28 (29)
Advice from parents or other authority figures	34 (28)
General internet searches and online information	61 (24)
Awareness of and interest in psychedelic research	
I am aware of this, and am well educated on the topic.	22 (16.7%)
I am aware of this, and curious to know more.	84 (63.6%)
I am aware of this, but it doesn't interest me.	3 (2.3%)
I am not aware of this, but am curious to know more.	22 (16.7%)
I am not aware of this, and it doesn't interest me.	1 (0.8%)
Perception of mental health treatments (0 [not effective] to 100 [extremely effective])	
Pharmacological (e.g., selective-serotonin reuptake inhibitors [SSRIs])	58.7 (19.7)
Psychotherapy	66.9 (20.9)
Openness to experience (BFI-10)	3.72 (0.92)
Conscientiousness (BFI-10)	3.76 (0.88)
Extraversion (BFI-10)	3.61 (0.98)
Agreeableness (BFI-10)	3.63 (0.86)
Neuroticism (BFI-10)	3.01 (1.14)

Abbreviations: SD, standard deviation; BFI-10, 10-item Big Five Inventory.

For peer review only

Table 2. Descriptive statistics of medical students' self-reported knowledge and frequency of encountering psychedelics in teaching and in extracurricular reading (N = 132).

	Self-reported knowledge [1]	Encountered in curriculum [2]	Encountered in extracurricular reading [2]
Cannabis	75.6 (17.7)	43.9 (28.4)	60.1 (26.5)
Cocaine	65.6 (22.2)	42.5 (27.5)	46.0 (28.4)
Heroin	60.2 (21.7)	39.5 (29.8)	41.3 (30.0)
Ketamine	61.2 (22.5)	32.2 (27.5)	41.9 (28.9)
LSD	56.1 (27.9)	16.8 (19.7)	39.8 (33.7)
Magic mushrooms	57.5 (29.7)	14.1 (19.9)	36.4 (33.0)
MDMA (ecstasy)	64.5 (23.4)	27.8 (25.5)	40.0 (31.1)

Note. All variables represent the mean (and standard deviation).

[1] Ranging from 0 (no knowledge) to 100 (excellent knowledge).

[2] Ranging from 0 (never) to 100 (very often).

For peer review only

Table 3. Multidimensional harm assessment of seven drugs (*N* = 132).

	Drug-specific mortality	Drug-related mortality	Drug-specific damage	Dependence	Drug-specific impairment of mental functioning	Drug-related impairment of mental functioning
Cannabis	13.3 (17.6)	38.2 (25.5)	34.9 (25.5)	48.5 (27.5)	58.4 (27.4)	48.9 (26.7)
Cocaine	70.8 (21.4)	66.4 (20.2)	73.5 (17.9)	80 (16.9)	64 (23.7)	68.4 (22.8)
Heroin	89.9 (14.3)	85.3 (16.9)	84.5 (15.7)	93.9 (11.9)	69.5 (25.5)	89.3 (13.8)
Ketamine	57.4 (27.7)	49.4 (25)	54.6 (24.8)	49.7 (25.8)	52.6 (25)	49.6 (24.9)
LSD	35.5 (28.7)	39.4 (28.1)	36.5 (28.9)	31.1 (26.5)	60.9 (26.1)	39.4 (27.3)
Magic mushrooms	28.1 (26.2)	32.3 (25.6)	29.4 (24.6)	27.2 (23.7)	55.2 (28)	36.5 (27.2)
MDMA (ecstasy)	63.5 (26.7)	53.7 (25.3)	55.9 (25.6)	48.1 (26.6)	59 (26.2)	48 (24.6)

Note. All values represent the mean (and 95% confidence interval) on a scale ranging from 0 (no harm) to 100 (severe harm).

For peer review only

Table 4. Multivariable linear regression models of the association with support for legal change (*N* = 132).

	Legal change composite scores [1]		
	Full model		
Explanatory variables (unstandardised)	Estimate	95% CI	*p*-value
Psychedelic knowledge	0.31	0.13 to 0.48	0.001
Psychedelic harm	-0.43	-0.66 to -0.21	<0.001
Course year (1 year)	2.82	0.16 to 5.48	0.038
Interest in psychiatry	-0.68	-4.01 to 2.65	0.687
Perception of pharmacological treatments	-0.01	-0.22 to 0.2	0.927
Perception of nonpharmacological treatments	-0.19	-0.38 to 0	0.051
Openness to experience	1.62	-2.4 to 5.64	0.426
Neuroticism	-0.10	-3.35 to 3.16	0.952
(Intercept)	77.53	48.43 to 106.64	<0.001
	Final prediction model		
Explanatory variables (unstandardised)	Estimate	95% CI	*p*-value
Psychedelic knowledge	0.31	0.14 to 0.48	<0.001
Psychedelic harm	-0.45	-0.65 to -0.24	<0.001
Course year (1 year)	3.00	0.4 to 5.59	0.024
Perception of nonpharmacological treatments	-0.18	-0.36 to 0	0.044
(Intercept)	79.83	59.26 to 100.40	<0.001

Abbreviations: CI, confidence interval.

[1] Ranging from 0 (strongly oppose) to 100 (strongly support).

[2] The final model retained all explanatory variables with $p < 0.05$.

For peer review only

Figure 1. Drugs ordered by their overall harm scores, showing sub-scores for each of the six harm criteria. Harm scores range from 0 (no harm) to 100 (severe harm). DSM = drug-specific mortality. DRM = drug-related mortality. DSD = drug-specific damage. DEP = dependence. DSI = drug-specific impairment of mental functioning. DRI = drug-related impairment of mental functioning. LSD = lysergic acid diethylamide.

For peer review only

For peer review only - http://bmjopen.bmj.com/site/about/guidelines.xhtml

Author contributions

Charlie Song-Smith: Conception of the work, Methodology, Data acquisition, Data analysis, Reviewing the work critically for important intellectual content.
Edward Jacobs: Conception of the work, Methodology, Reviewing the work critically for important intellectual content, Supervision.
James Rucker: Conception of the work, Reviewing the work critically for important intellectual content.
Matthew Saint: Conception of the work, Reviewing the work critically for important intellectual content.
James Cooke: Conception of the work, Methodology, Reviewing the work critically for important intellectual content, Supervision.
Marco Schlosser: Writing of the original draft, Conception of the work, Methodology, Data analysis, Data interpretation, Visualisation, Supervision.

Competing interests statement: James Rucker has received payments for advisory work from Clerkenwell Health (Past), Beckley PsyTech (Past), Delica Therapeutics (Past), for articles written for Janssen, and financial assistance for attendance at conferences from Compass Pathways (past) and Janssen. James Rucker has received grant funding (received and managed by King's College London) from Compass Pathfinder, Beckley PsyTech, Multidisciplinary Association for Psychedelic Studies, National Institute for Health Research, Wellcome Trust, Biomedical Research Centre at the South London and Maudsley NHS Foundation Trust. All other authors declare that they have no competing interests.

Funding statement: This research was funded in whole, or in part, by the Wellcome Trust 221152/Z/20/Z. For the purpose of Open Access, the author has applied a CC BY public copyright licence to any Author Accepted Manuscript version arising from this submission. James Rucker received salary support via the NIHR Clinician Scientist Fellowship Programme during the period of this research (CS-2017-17-007).

Data availability statement: All data and analysis scripts can be found on the senior author's Open Science Framework profile: https://osf.io/tmp3u/.

Ethics approval: This study received ethical approval from University College London's Research Ethics Committee (Project ID: 14309/007) and was performed in accordance with the 1964 Declaration of Helsinki and its later amendments.

For peer review only - http://bmjopen.bmj.com/site/about/guidelines.xhtml

References

1. Carhart-Harris RL, Goodwin GM. The Therapeutic Potential of Psychedelic Drugs: Past, Present, and Future. Neuropsychopharmacology. 2017 Oct;42(11):2105–13.

2. Nichols DE. Psychedelics. Barker EL, editor. Pharmacol Rev. 2016 Apr 1;68(2):264–355.

3. Nutt D, Erritzoe D, Carhart-Harris R. Psychedelic Psychiatry's Brave New World. Cell. 2020 Apr 2;181(1):24–8.

4. Rucker JJH, Iliff J, Nutt DJ. Psychiatry & the psychedelic drugs. Past, present & future. Neuropharmacology. 2018 Nov 1;142:200–18.

5. Nichols DE, Walter H. The History of Psychedelics in Psychiatry. Pharmacopsychiatry. 2021 Jul;54(4):151–66.

6. Nichols D, Johnson M, Nichols C. Psychedelics as Medicines: An Emerging New Paradigm. Clin Pharmacol Ther. 2017;101(2):209–19.

7. Andersen KAA, Carhart-Harris R, Nutt DJ, Erritzoe D. Therapeutic effects of classic serotonergic psychedelics: A systematic review of modern-era clinical studies. Acta Psychiatr Scand. 2021;143(2):101–18.

8. Reiff CM, Richman EE, Nemeroff CB, Carpenter LL, Widge AS, Rodriguez CI, et al. Psychedelics and Psychedelic-Assisted Psychotherapy. Am J Psychiatry. 2020 May;177(5):391–410.

9. Johnson MW, Hendricks PS, Barrett FS, Griffiths RR. Classic psychedelics: An integrative review of epidemiology, therapeutics, mystical experience, and brain network function. Pharmacol Ther. 2019 May 1;197:83–102.

10. Aday JS, Mitzkovitz CM, Bloesch EK, Davoli CC, Davis AK. Long-term effects of psychedelic drugs: A systematic review. Neurosci Biobehav Rev. 2020 Jun 1;113:179–89.

11. Carhart-Harris R, Giribaldi B, Watts R, Baker-Jones M, Murphy-Beiner A, Murphy R, et al. Trial of Psilocybin versus Escitalopram for Depression. N Engl J Med. 2021 Apr 15;384(15):1402–11.

12. Raison CL, Sanacora G, Woolley J, Heinzerling K, Dunlop BW, Brown RT, et al. Single-Dose Psilocybin Treatment for Major Depressive Disorder: A Randomized Clinical Trial. JAMA. 2023 Sep 5;330(9):843–53.

13. Rotz R von, Schindowski EM, Jungwirth J, Schuldt A, Rieser NM, Zahoranszky K, et al. Single-dose psilocybin-assisted therapy in major depressive disorder: a placebo-controlled, double-blind, randomised clinical trial. eClinicalMedicine [Internet]. 2023 Feb 1 [cited 2023 Nov 29];56. Available from: https://www.thelancet.com/journals/eclinm/article/PIIS2589-5370(22)00538-7/fulltext?trk=public_post_reshare_feed-article-content

For peer review only

14. Goodwin GM, Aaronson ST, Alvarez O, Atli M, Bennett JC, Croal M, et al. Single-dose psilocybin for a treatment-resistant episode of major depression: Impact on patient-reported depression severity, anxiety, function, and quality of life. J Affect Disord. 2023 Apr 14;327:120–7.

15. Mertens LJ, Koslowski M, Betzler F, Evens R, Gilles M, Jungaberle A, et al. Methodological challenges in psychedelic drug trials: Efficacy and safety of psilocybin in treatment-resistant major depression (EPIsoDE) – Rationale and study design. Neurosci Appl. 2022 Jan 1;1:100104.

16. Mitchell JM, Bogenschutz M, Lilienstein A, Harrison C, Kleiman S, Parker-Guilbert K, et al. MDMA-assisted therapy for severe PTSD: a randomized, double-blind, placebo-controlled phase 3 study. Nat Med. 2021 Jun;27(6):1025–33.

17. Illingworth BJ, Lewis DJ, Lambarth AT, Stocking K, Duffy JM, Jelen LA, et al. A comparison of MDMA-assisted psychotherapy to non-assisted psychotherapy in treatment-resistant PTSD: A systematic review and meta-analysis. J Psychopharmacol (Oxf). 2021 May 1;35(5):501–11.

18. aan het Rot M, Zarate CA, Charney DS, Mathew SJ. Ketamine for Depression: Where Do We Go from Here? Biol Psychiatry. 2012 Oct 1;72(7):537–47.

19. Meshkat S, Haikazian S, Di Vincenzo JD, Fancy F, Johnson D, Chen-Li D, et al. Oral ketamine for depression: An updated systematic review. World J Biol Psychiatry. 2023 Aug 9;24(7):545–57.

20. Xu Y, Hackett M, Carter G, Loo C, Gálvez V, Glozier N, et al. Effects of Low-Dose and Very Low-Dose Ketamine among Patients with Major Depression: a Systematic Review and Meta-Analysis. Int J Neuropsychopharmacol. 2016 Apr 1;19(4):pyv124.

21. Aday JS, Carhart-Harris RL, Woolley JD. Emerging Challenges for Psychedelic Therapy. JAMA Psychiatry. 2023 Jun 1;80(6):533–4.

22. Aday JS, Barnett BS, Grossman D, Murnane KS, Nichols CD, Hendricks PS. Psychedelic Commercialization: A Wide-Spanning Overview of the Emerging Psychedelic Industry. Psychedelic Med. 2023 Sep;1(3):150–65.

23. Barnett BS, Beaussant Y, King F, Doblin R. Psychedelic Knowledge and Opinions in Psychiatrists at Two Professional Conferences: An Exploratory Survey. J Psychoactive Drugs. 2022 May 27;54(3):269–77.

24. Barnett BS, Siu WO, Pope HGJ. A Survey of American Psychiatrists' Attitudes Toward Classic Hallucinogens. J Nerv Ment Dis. 2018 Jun;206(6):476.

25. Barnett BS, Arakelian M, Beebe D, Ontko J, Riegal C, Siu WO, et al. American Psychiatrists' Opinions About Classic Hallucinogens and Their Potential Therapeutic Applications: A 7-Year Follow-Up Survey. Psychedelic Med [Internet]. 2023 Sep 19 [cited 2024 Feb 9]; Available from: https://www.liebertpub.com/doi/abs/10.1089/psymed.2023.0036

26. Page LA, Rehman A, Syed H, Forcer K, Campbell G. The Readiness of Psychiatrists to Implement Psychedelic-Assisted Psychotherapy. Front Psychiatry [Internet]. 2021 [cited

For peer review only

2023 Nov 29];12. Available from: https://www.frontiersin.org/articles/10.3389/fpsyt.2021.743599

27. Davis AK, Agin-Liebes G, España M, Pilecki B, Luoma J. Attitudes and Beliefs about the Therapeutic Use of Psychedelic Drugs among Psychologists in the United States. J Psychoactive Drugs. 2022 Aug 8;54(4):309–18.

28. Hearn BG, Brubaker MD, Richardson G. Counselors' attitudes toward psychedelics and their use in therapy. J Couns Dev. 2022;100(4):364–73.

29. Wang K, Sun Y, Nava B, Sampiere L, Jacobs RJ. Predictors of Medical Students' Perceptions of Psilocybin-Assisted Therapy for Use in Medical Practice. Cureus. 2023;15(4):e37450.

30. Li I, Fong R, Hagen M, Tabaac B. Medical student attitudes and perceptions of psychedelic-assisted therapies. Front Psychiatry. 2023 Jun 27;14:1190507.

31. Pagán AF, Lex C, Soares JC, Meyer TD. Medical Students' Attitudes and Beliefs Toward Psilocybin: Does Terminology and Personal Experience with Psychedelics Matter? Psychedelic Med. 2023 Sep;1(3):130–8.

32. Cheeta S, Halil A, Kenny M, Sheehan E, Zamyadi R, Williams AL, et al. Does perception of drug-related harm change with age? A cross-sectional online survey of young and older people. BMJ Open. 2018 Nov 1;8(11):e021109.

33. Nutt DJ, King LA, Phillips LD. Drug harms in the UK: a multicriteria decision analysis. The Lancet. 2010 Nov 6;376(9752):1558–65.

34. Prohibition Partners. PSYCH: The Psychedelics as Medicine Report Second Edition. 2020.

35. Rammstedt B, John OP. Measuring personality in one minute or less: A 10-item short version of the Big Five Inventory in English and German. J Res Personal. 2007 Feb 1;41(1):203–12.

36. John OP, Srivastava S. The Big Five Trait taxonomy: History, measurement, and theoretical perspectives. In: Handbook of personality: Theory and research, 2nd ed. New York, NY, US: Guilford Press; 1999. p. 102–38.

37. Kopra EI, Ferris JA, Winstock AR, Kuypers KP, Young AH, Rucker JJ. Investigation of self-treatment with lysergic acid diethylamide and psilocybin mushrooms: Findings from the Global Drug Survey 2020. J Psychopharmacol Oxf Engl. 2023 Jul;37(7):733–48.

38. Walsh CA, Gorfinkel L, Shmulewitz D, Stohl M, Hasin DS. Use of Lysergic Acid Diethylamide by Major Depression Status. JAMA Psychiatry. 2024 Jan 1;81(1):89–96.

39. Boehnke KF, Cox K, Weston C, Herberholz M, Glynos N, Kolbman N, et al. Slouching towards engagement: interactions between people using psychedelics naturalistically and their healthcare providers. Front Psychiatry. 2023;14:1224551.

40. Nayak SM, Gukasyan N, Barrett FS, Erowid E, Erowid F, Griffiths RR. Classic Psychedelic Coadministration with Lithium, but Not Lamotrigine, is Associated with

For peer review only

Seizures: An Analysis of Online Psychedelic Experience Reports. Pharmacopsychiatry. 2021 Sep;54(5):240–5.

41. Aday JS, Skiles Z, Eaton N, Fredenburg L, Pleet M, Mantia J, et al. Personal Psychedelic Use Is Common Among a Sample of Psychedelic Therapists: Implications for Research and Practice. Psychedelic Med. 2023 Mar;1(1):27–37.

[dataset] 42. Song-Smith C, Jacobs E, Rucker J, Saint M, Cooke J, Schlosser M. UK medical students' self-reported knowledge and harm assessment of psychedelics and their application in clinical research: a cross-sectional study. December 21, 2023. Open Science Framework. https://doi.org/10.17605/OSF.IO/TMP3U

For peer review only

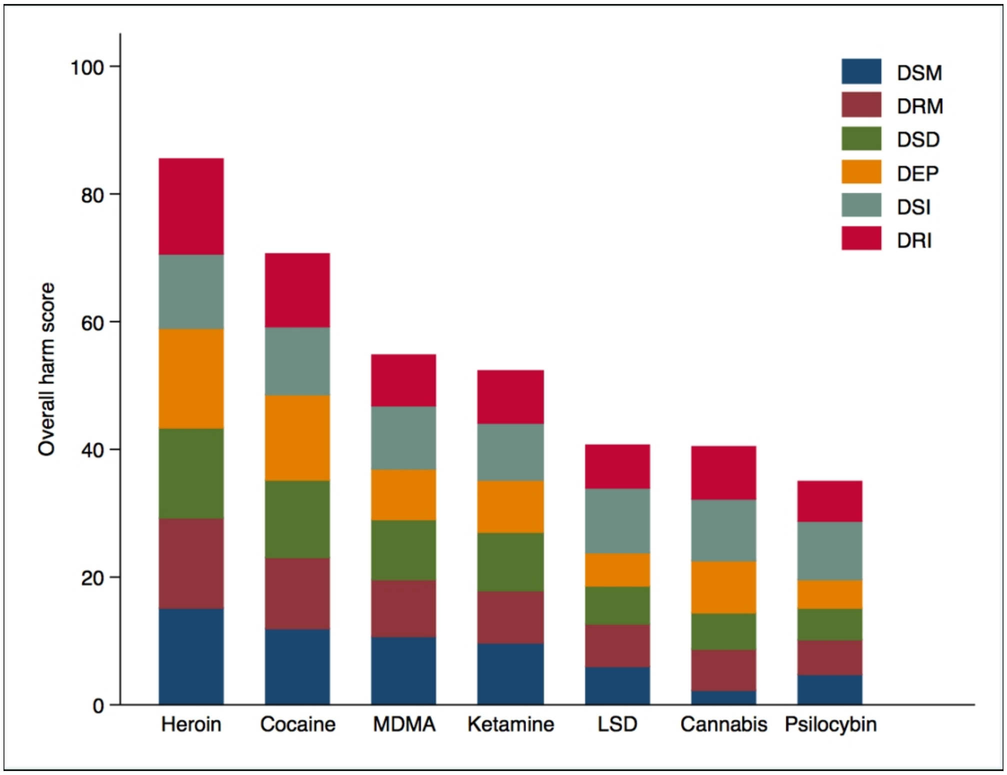

Figure 1. Drugs ordered by their overall harm scores, showing sub-scores for each of the six harm criteria. Harm scores range from 0 (no harm) to 100 (severe harm). DSM = drug-specific mortality. DRM = drug-related mortality. DSD = drug-specific damage. DEP = dependence. DSI = drug-specific impairment of mental functioning. DRI = drug-related impairment of mental functioning. LSD = lysergic acid diethylamide.

222x170mm (144 x 144 DPI)

Medical Student Drugs and Mental Health Survey

Start of Block: Participant Information

Q1 **Medical Students' Knowledge and Perceptions of Common "Recreational" Drugs and Their Potential Clinical Uses**

Study Purpose
The study aims to investigate the following:
- Medical students' knowledge and perceptions of commonly used "recreational" drugs.
- Students' perspectives on the current and future clinical uses of these drugs, especially in the treatment of some mental health conditions.
- Factors that influence students' perception of drugs.
The study is led by Marco Schlosser in the UCL Division of Psychiatry and Charlie Song-Smith (BSc), an undergraduate on the MBBS programme at UCL.

Who can participate?
To take part you must be at least 18 years old, have a good understanding of the English language, and be a current student enrolled in a UK medical (MBBS) programme accredited by the General Medical Council (GMC). Students who are currently undertaking an intercalated BSc or other degree as part of their programme (including MBPhD students) are eligible to participate.

What will participation involve?
This study uses an anonymous online survey that approximately 10 minutes to complete. The survey will consist of several brief sections asking about your knowledge, perceptions and some personal factors. After completing the survey, you will have no further involvement and will not be contacted again. To improve the reliability and transparency of scientific research, we will make the fully anonymous data we collect in this study freely available online for other researchers. We greatly appreciate the time you dedicate to this project.

Do I have to participate?
It is up to you to decide whether to take part or not; choosing not to take part will not disadvantage you in any way. If you do decide to take part, you are free to withdraw at any time without giving a reason, by simply closing the browser before the end of the survey. Please note that fully completed surveys cannot be removed as we will be unable to selectively identify and delete your responses from the pool of anonymised data.

What are the possible benefits of taking part?

For peer review only

For peer review only - http://bmjopen.bmj.com/site/about/guidelines.xhtml

The data we collect from this study will shed light on how well educated and knowledgeable medical students feel about commonly used drugs, and will therefore help to inform medical education. The study results will also help to gauge students' views on the risks and clinical uses of drugs in relation to mental health, which has potential to influence public policy and future clinical practice. You may personally benefit from an increased knowledge of the various harms of different drugs, as well as increased awareness of the ethical and scientific questions surrounding the clinical uses of some drugs.

Are there any risks of taking part?
The risks to taking part in this study are minimal. Though the survey may touch on sensitive issues concerning illicit drugs, we will not ask you for any information relating to personal use of drugs by yourself or others, nor any other illegal or inappropriate activities. You may feel strongly about your responses, but can rest assured that they may not be linked to you in any way. If you feel distressed or upset by anything in the survey, we encourage you to report this to the researchers via the following:
Mr Marco Schlosser (MSc): marco.schlosser@ucl.ac.uk
Mr Charlie Song-Smith (BSc): charlie.song-smith.19@ucl.ac.uk

Data Protection Privacy Notice
The data you provide is very valuable to us. No personally identifying information will be collected and we will not record your IP address. All obtained data are anonymous. Hence, the publication of study results will not include any data that can directly or indirectly identify you. Your responses will be stored securely by UCL for the duration of the research, putatively for a period of 5 years. University College London (UCL) will act as the data controller for this project. This study constitutes social research which is carried out by the university in the public interest, and your data will be processed in compliance with the UK General Data Protection Regulation (GDPR). This study has received full ethical approval from UCL's Research Ethics Committee (Project ID: 14309/007).

Study results
The results of the study will be analysed, written up and submitted to a peer-reviewed scientific journal. If you would like to know about the results upon completion of the research, irrespective of whether you partake in this study, please follow Marco Schlosser's Open Science Framework profile, which will make all the data and results freely available online: https://osf.io/wgejf/

Thank you for reading this information sheet and for considering whether to take part in this research. Please check the boxes below to confirm that you have read and understood this information sheet and provide your informed consent to participate.

I understand that I am free to withdraw from the study before the completion of the survey. (1)	○ Confirm (1)

For peer review only - http://bmjopen.bmj.com/site/about/guidelines.xhtml

I understand that my anonymous responses will be stored securely by UCL for 5 years. (2)	○ Confirm (1)
I am at least 18 years old and have a good understanding of the English language. (3)	○ Confirm (1)
I am currently enrolled as a medical student in a GMC-accredited programme, and I voluntarily consent to participate in this study. (4)	○ Confirm (1)

Skip To: End of Survey If Q1 != I am currently enrolled as a medical student in a GMC-accredited programme, and I voluntarily consent to participate in this study. [Answer 1]

End of Block: Participant Information

Start of Block: Self-reported drug knowledge

Display This Question:

If Q1 = I understand that I am free to withdraw from the study before the completion of the survey. [Answer 1]

And Q1 = I understand that my anonymous responses will be stored securely by UCL for 5 years. [Answer 1]

And Q1 = I am at least 18 years old and have a good understanding of the English language. [Answer 1]

And Q1 = I am currently enrolled as a medical student in a GMC-accredited programme, and I voluntarily consent to participate in this study. [Answer 1]

Q1 Thank you for participating in this survey. Most questions require a single click or tap (if using mobile/tablet) on a sliding visual analogue scale to indicate your response. You are able to move the slider backwards and forwards to fine tune your preferences.

The first section of this questionnaire will focus on your knowledge and perceptions of the following commonly used "recreational" (and illegal) drugs: cannabis, cocaine, heroin, LSD, MDMA (ecstasy) and psilocybin (magic mushrooms).

Firstly, please indicate how well educated/knowledgeable you feel about these drugs.
For example, this would include knowledge of pharmacology, physiology, subjective effects and adverse consequences of the drug.

If you have not heard of the drug in question, please select the checkbox.

	No knowledge	Excellent knowledge	Not heard of it

For peer review only - http://bmjopen.bmj.com/site/about/guidelines.xhtml

Cannabis ()	
Cocaine ()	
Heroin ()	
Ketamine ()	
LSD ()	
Magic mushrooms ()	
MDMA (ecstasy) ()	

Q2 How often have you come across the drugs listed above in your teaching, including lectures, small group work and problem-based or self-directed learning?

This excludes any direct observation of patients/members of the public, e.g. on clinical placements.

	Never ... Very often
Cannabis ()	
Cocaine ()	
Heroin ()	
Ketamine ()	
LSD ()	
Magic mushrooms ()	
MDMA (ecstasy) ()	

For peer review only

Q3 How often have you come across these drugs in your own reading/research?

	Never	Very often
Cannabis ()		
Cocaine ()		
Heroin ()		
Ketamine ()		
LSD ()		
Magic mushrooms ()		
MDMA (ecstasy) ()		

For peer review only

End of Block: Self-reported drug knowledge

Start of Block: Perception of drug-related harms

Q4

The following section will ask you to rate a number of different harms associated with the drugs listed, from no harm to severe harm.

When considering these harms, please make your judgments on the assumption that the drugs are pure, i.e. not contaminated with other substances, and taken in isolation. For example, try your best to rate the harms of pure MDMA that is not cut with other drugs, or intentionally mixed with other drugs by the user.

How great is the potential of the drug to cause death by overdose (i.e. the intrinsic lethality)?

No harm Severe harm

0 10 20 30 40 50 60 70 80 90 100

For peer review only - http://bmjopen.bmj.com/site/about/guidelines.xhtml

Cannabis ()	
Cocaine ()	
Heroin ()	
Ketamine ()	
LSD ()	
Magic mushrooms ()	
MDMA (ecstasy) ()	

For peer review only

Q5

To what extent is life is shortened by the use of the drug, excluding deaths from overdose? For example, this could include deaths due to road traffic accidents, lung cancers, HIV and suicide.

	No harm									Severe harm
0	10	20	30	40	50	60	70	80	90	100

Cannabis ()	
Cocaine ()	
Heroin ()	
Ketamine ()	
LSD ()	
Magic mushrooms ()	
MDMA (ecstasy) ()	

Q6
How great is the damage to physical health (short of death) caused by the drug? For example, this could include liver damage, other organ damage, seizures, strokes.

No harm Severe harm

0 10 20 30 40 50 60 70 80 90 100

Cannabis ()

Cocaine ()

Heroin ()

Ketamine ()

LSD ()

Magic mushrooms ()

MDMA (ecstasy) ()

Q7
To what extent does the drug cause addiction/dependence, i.e. the urge to continue to use despite adverse consequences?

No harm Severe harm

0 10 20 30 40 50 60 70 80 90 100

For peer review only

Cannabis ()	
Cocaine ()	
Heroin ()	
Ketamine ()	
LSD ()	
Magic mushrooms ()	
MDMA (ecstasy) ()	

For peer review only

Q8
To what extent does the drug cause acute mental health problems when taken?

	No harm	Severe harm
	0 10 20 30 40 50 60 70 80 90 100	
Cannabis ()		
Cocaine ()		
Heroin ()		
Ketamine ()		
LSD ()		
Magic mushrooms ()		
MDMA (ecstasy) ()		

For peer review only - http://bmjopen.bmj.com/site/about/guidelines.xhtml

Q9 To what extent does the drug cause problems in the user's work or social life, leading to mental health problems (excluding addiction)?

	No harm ... Severe harm<br>0 10 20 30 40 50 60 70 80 90 100
Cannabis ()	
Cocaine ()	
Heroin ()	
Ketamine ()	
LSD ()	
Magic mushrooms ()	
MDMA (ecstasy) ()	

For peer review only

End of Block: Perception of drug-related harms

Start of Block: Drugs in psychiatry

Q10
This short section will ask you for your perceptions of current psychiatric therapies and novel drug treatments.

Thinking about mental health conditions such as anxiety disorders, depression and addiction, how effective would you consider the following general treatment approaches for these conditions?

	Not effective at all ... Extremely effective
Pharmaceuticals, e.g. selective-serotonin reuptake inhibitors (SSRIs) ()	
Psychotherapy, e.g. cognitive behavioural therapy (CBT) or counselling ()	

For peer review only - http://bmjopen.bmj.com/site/about/guidelines.xhtml

Q11 Consider the following statement: “Drugs such as psilocybin (the active ingredient in magic mushrooms) and MDMA (the active ingredient in "ecstasy") have recently been investigated as potential treatments for mental health conditions such as depression, PTSD and addiction.”

Which of the following best describes your own awareness of this research area?

- ◯ I am aware of this, and am well educated on the topic (1)
- ◯ I am aware of this, and curious to know more (2)
- ◯ I am aware of this, but it doesn't interest me (3)
- ◯ I am not aware of this, but am curious to know more (4)
- ◯ I am not aware of this, and it doesn't interest me (5)

Q12 Consider the following statement: "Drugs that are considered to have no medical value and a high risk of misuse or harm are listed in Schedule 1 of the UK Misuse of Drugs Regulations (2001); these currently include MDMA and psilocybin. Research involving schedule 1 drugs is associated with significantly increased costs, duration and difficulty."

Given your current knowledge, to what extent would you support or oppose changing the legal status of these drugs to facilitate further research into their potential medical uses (while keeping restrictions on recreational use as they are)?

	Strongly oppose Strongly support
Psilocybin ()	
MDMA ()	

End of Block: Drugs in psychiatry

Start of Block: Personal factors

Q13
This final section will ask you about some personal factors, including a personality measure and the factors which have influenced your perception of drugs. We appreciate that these questions

For peer review only - http://bmjopen.bmj.com/site/about/guidelines.xhtml

may touch on sensitive issues and greatly appreciate your responses.

What year of your course are you in currently?

- 1 (1)
- 2 (2)
- 3 (3)
- 4 (4)
- 5 (5)
- 6 (6)

Q14 Have you previously or are you currently undertaking an intercalated BSc or MBPhD as part of your degree programme?

- Yes (1)
- No (2)

Q15 Have you previously completed a degree (BA/BSc, MSci, PhD, etc) outside of your current programme?

- Yes (1)
- No (2)

For peer review only

For peer review only - http://bmjopen.bmj.com/site/about/guidelines.xhtml

Q16 To what extent are you interested in pursuing a career in psychiatry?

- ◯ I am confident I will pursue a career in psychiatry (1)
- ◯ I consider psychiatry one of my more likely career paths (2)
- ◯ I have not ruled out psychiatry, but it is not one of my more likely career paths (3)
- ◯ I consider psychiatry one of my less likely career paths (4)
- ◯ I am confident I will not pursue a career in psychiatry (5)

Q17 Please rate the importance of the following factors in influencing your perception of drugs (such as those listed in this survey).

	Not important — Very important<br>0 10 20 30 40 50 60 70 80 90 100
My own and my friends' personal experiences ()	
Teaching as part of my course ()	
Media reporting ()	
Reading scientific papers/articles ()	
A drug's legal class (A, B, C) ()	
Advice from parents or other authority figures ()	
General internet searches and online information ()	

For peer review only - http://bmjopen.bmj.com/site/about/guidelines.xhtml

Q18 Finally, please select how well you feel the following statements describe your personality.

I see myself as someone who...

	Disagree strongly (1)	Disagree a little (2)	Neither agree nor disagree (3)	Agree a little (4)	Agree strongly (5)
is reserved (1)	○	○	○	○	○
is generally trusting (2)	○	○	○	○	○
tends to be lazy (3)	○	○	○	○	○
is relaxed, handles stress well (4)	○	○	○	○	○
has few artistic interests (5)	○	○	○	○	○
is outgoing, sociable (6)	○	○	○	○	○
tends to find fault with others (7)	○	○	○	○	○
does a thorough job (8)	○	○	○	○	○
gets nervous easily (9)	○	○	○	○	○
has an active imagination (10)	○	○	○	○	○

End of Block: Personal factors

For peer review only

Supplement 3: Checklist for Reporting Of Survey Studies (CROSS)

Section/topic	Item	Item description	Reported on page #
**Title and abstract**			
Title and abstract	1a	State the word "survey" along with a commonly used term in title or abstract to introduce the study's design.	2
	1b	Provide an informative summary in the abstract, covering background, objectives, methods, findings/results, interpretation/discussion, and conclusions.	2
**Introduction**			
Background	2	Provide a background about the rationale of study, what has been previously done, and why this survey is needed.	4-6
Purpose/aim	3	Identify specific purposes, aims, goals, or objectives of the study.	6
**Methods**			
Study design	4	Specify the study design in the methods section with a commonly used term (e.g., cross-sectional or longitudinal).	7
Data collection methods	5a	Describe the questionnaire (e.g., number of sections, number of questions, number and names of instruments used).	8
	5b	Describe all questionnaire instruments that were used in the survey to measure particular concepts. Report target population, reported validity and reliability information, scoring/classification procedure, and reference links (if any).	8-11
	5c	Provide information on pretesting of the questionnaire, if performed (in the article or in an online supplement). Report the method of pretesting, number of times questionnaire was pre-tested, number and demographics of participants used for pretesting, and the level of similarity of demographics between pre-testing participants and sample population.	n/a
	5d	Questionnaire if possible, should be fully provided (in the article, or as appendices or as an online supplement).	Online Suppl.
Sample characteristics	6a	Describe the study population (i.e., background, locations, eligibility criteria for participant inclusion in survey, exclusion criteria).	8
	6b	Describe the sampling techniques used (e.g., single stage or multistage sampling, simple random sampling, stratified sampling, cluster sampling, convenience sampling). Specify the locations of sample participants whenever clustered sampling was applied.	7
	6c	Provide information on sample size, along with details of sample size calculation.	12
	6d	Describe how representative the sample is of the study population (or target population if possible), particularly for population-based surveys.	7
Survey	7a	Provide information on modes of questionnaire administration, including the type and number of contacts, the location where the survey was conducted (e.g., outpatient	7

For peer review only

administration		room or by use of online tools, such as SurveyMonkey).	
	7b	Provide information of survey's time frame, such as periods of recruitment, exposure, and follow-up days.	7
	7c	Provide information on the entry process:<br>–>For non-web-based surveys, provide approaches to minimize human error in data entry.<br>–>For web-based surveys, provide approaches to prevent "multiple participation" of participants.	7
Study preparation	8	Describe any preparation process before conducting the survey (e.g., interviewers' training process, advertising the survey).	7
Ethical considerations	9a	Provide information on ethical approval for the survey if obtained, including informed consent, institutional review board [IRB] approval, Helsinki declaration, and good clinical practice [GCP] declaration (as appropriate).	7
	9b	Provide information about survey anonymity and confidentiality and describe what mechanisms were used to protect unauthorized access.	7
Statistical analysis	10a	Describe statistical methods and analytical approach. Report the statistical software that was used for data analysis.	12-13
	10b	Report any modification of variables used in the analysis, along with reference (if available).	n/a
	10c	Report details about how missing data was handled. Include rate of missing items, missing data mechanism (i.e., missing completely at random [MCAR], missing at random [MAR] or missing not at random [MNAR]) and methods used to deal with missing data (e.g., multiple imputation).	8
	10d	State how non-response error was addressed.	8
	10e	For longitudinal surveys, state how loss to follow-up was addressed.	n/a
	10f	Indicate whether any methods such as weighting of items or propensity scores have been used to adjust for non-representativeness of the sample.	n/a
	10g	Describe any sensitivity analysis conducted.	n/a
**Results**			
Respondent characteristics	11a	Report numbers of individuals at each stage of the study. Consider using a flow diagram, if possible.	n/a
	11b	Provide reasons for non-participation at each stage, if possible.	8
	11c	Report response rate, present the definition of response rate or the formula used to calculate response rate.	13

For peer review only

	11d	Provide information to define how unique visitors are determined. Report number of unique visitors along with relevant proportions (e.g., view proportion, participation proportion, completion proportion).	n/a
Descriptive results	12	Provide characteristics of study participants, as well as information on potential confounders and assessed outcomes.	13
Main findings	13a	Give unadjusted estimates and, if applicable, confounder-adjusted estimates along with 95% confidence intervals and p-values.	13-14
	13b	For multivariable analysis, provide information on the model building process, model fit statistics, and model assumptions (as appropriate).	14
	13c	Provide details about any sensitivity analysis performed. If there are considerable amount of missing data, report sensitivity analyses comparing the results of complete cases with that of the imputed dataset (if possible).	n/a
**Discussion**			
Limitations	14	Discuss the limitations of the study, considering sources of potential biases and imprecisions, such as non-representativeness of sample, study design, important uncontrolled confounders.	17
Interpretations	15	Give a cautious overall interpretation of results, based on potential biases and imprecisions and suggest areas for future research.	15-16
Generalizability	16	Discuss the external validity of the results.	18
**Other sections**			
Role of funding source	17	State whether any funding organization has had any roles in the survey's design, implementation, and analysis.	1
Conflict of interest	18	Declare any potential conflict of interest.	1
Acknowledgements	19	Provide names of organizations/persons that are acknowledged along with their contribution to the research.	n/a
